# Key Metabolites and Mechanistic Changes for Salt Tolerance in an Experimentally Evolved Sulfate-Reducing Bacterium, *Desulfovibrio vulgaris*

**DOI:** 10.1128/mBio.01780-17

**Published:** 2017-11-14

**Authors:** Aifen Zhou, Rebecca Lau, Richard Baran, Jincai Ma, Frederick von Netzer, Weiling Shi, Drew Gorman-Lewis, Megan L. Kempher, Zhili He, Yujia Qin, Zhou Shi, Grant M. Zane, Liyou Wu, Benjamin P. Bowen, Trent R. Northen, Kristina L. Hillesland, David A. Stahl, Judy D. Wall, Adam P. Arkin, Jizhong Zhou

**Affiliations:** a Institute for Environmental Genomics, Department of Microbiology and Plant Biology, University of Oklahoma, Norman, Oklahoma, USA; b Environmental Genomics and Systems Biology Division, Lawrence Berkeley National Laboratory, Berkeley, California, USA; c Department of Civil and Environmental Engineering, University of Washington, Seattle, Washington, USA; d Department of Earth and Space Sciences, University of Washington, Seattle, Washington, USA; e Departments of Biochemistry and Molecular Microbiology and Immunology, University of Missouri—Columbia, Columbia, Missouri, USA; f School of Science, Technology, Engineering and Mathematics, University of Washington Bothell, Bothell, Washington, USA; g Department of Bioengineering, University of California Berkeley, Berkeley, California, USA; h Earth Sciences Division, Lawrence Berkeley National Laboratory, Berkeley, California, USA; i State Key Joint Laboratory of Environment Simulation and Pollution Control, School of Environment, Tsinghua University, Beijing, China; Max Planck Institute for Marine Microbiology

**Keywords:** *Desulfovibrio vulgaris*, PLFA, cell motility, energy efficiency, genomic mutations, organic solutes, transcriptomics

## Abstract

Rapid genetic and phenotypic adaptation of the sulfate-reducing bacterium *Desulfovibrio vulgaris* Hildenborough to salt stress was observed during experimental evolution. In order to identify key metabolites important for salt tolerance, a clone, ES10-5, which was isolated from population ES10 and allowed to experimentally evolve under salt stress for 5,000 generations, was analyzed and compared to clone ES9-11, which was isolated from population ES9 and had evolved under the same conditions for 1,200 generations. These two clones were chosen because they represented the best-adapted clones among six independently evolved populations. ES10-5 acquired new mutations in genes potentially involved in salt tolerance, in addition to the preexisting mutations and different mutations in the same genes as in ES9-11. Most basal abundance changes of metabolites and phospholipid fatty acids (PLFAs) were lower in ES10-5 than ES9-11, but an increase of glutamate and branched PLFA i17:1ω9c under high-salinity conditions was persistent. ES9-11 had decreased cell motility compared to the ancestor; in contrast, ES10-5 showed higher cell motility under both nonstress and high-salinity conditions. Both genotypes displayed better growth energy efficiencies than the ancestor under nonstress or high-salinity conditions. Consistently, ES10-5 did not display most of the basal transcriptional changes observed in ES9-11, but it showed increased expression of genes involved in glutamate biosynthesis, cation efflux, and energy metabolism under high salinity. These results demonstrated the role of glutamate as a key osmolyte and i17:1ω9c as the major PLFA for salt tolerance in *D. vulgaris*. The mechanistic changes in evolved genotypes suggested that growth energy efficiency might be a key factor for selection.

## IMPORTANCE

High salinity (e.g., elevated NaCl) is a stressor that affects many organisms. Salt tolerance, a complex trait involving multiple cellular pathways, is attractive for experimental evolutionary studies. *Desulfovibrio vulgaris* Hildenborough is a model sulfate-reducing bacterium (SRB) that is important in biogeochemical cycling of sulfur, carbon, and nitrogen, potentially for bio-corrosion, and for bioremediation of toxic heavy metals and radionuclides. The coexistence of SRB and high salinity in natural habitats and heavy metal-contaminated field sites laid the foundation for the study of salt adaptation of *D. vulgaris* Hildenborough with experimental evolution. Here, we analyzed a clone that evolved under salt stress for 5,000 generations and compared it to a clone evolved under the same condition for 1,200 generations. The results indicated the key roles of glutamate for osmoprotection and of i17:1ω9c for increasing membrane fluidity during salt adaptation. The findings provide valuable insights about the salt adaptation mechanism changes during long-term experimental evolution.

## INTRODUCTION

Microbial experimental evolution coupled with whole-genome sequencing has been widely used to identify key functional genes involved in desired traits, to improve the industrial efficiencies of microbes, and to address fundamental evolutionary questions (1–9). However, most evolutionary studies have focused on the endpoint strains or temporal transcriptional changes in evolved microorganisms. Very little is known about the changes of cellular components, such as metabolites and phospholipid fatty acids (PLFAs) during long-term evolution. Metabolites and PLFAs, as the end products of gene transcription, translation, and regulation, constitute the mechanistic basis for natural selection. Study of the temporal changes of these end products in experimentally evolved microorganisms may provide meaningful insights for the understanding of the adaptation mechanisms and also may inspire new theoretical directions (10).

Salinity (e.g., elevated NaCl) is an environmental factor that affects many organisms. The well-known strategies of microorganisms for coping with salt stress include intracellular accumulation of organic solutes and changes of membrane PLFA composition to compensate for the membrane fluidity decrease (11–13). *Desulfovibrio vulgaris* Hildenborough has been used as a model sulfate-reducing bacterium (SRB) for studying the complex physiology and stress responses (14) due to its importance in biogeochemical cycling of sulfur, carbon, and nitrogen and its potential to remediate toxic heavy metal contamination. Like *Escherichia coli* (15), *D. vulgaris* employs different mechanisms to combat salt stress, namely, an acute (16) or chronic response (17), depending on when the stress is encountered. In order to understand the salt adaptation mechanisms in *D. vulgaris* Hildenborough in an evolutionary context, six *D. vulgaris* Hildenborough replicate populations (ES7 to ES12) have been propagated under a mild salt stress condition. Analysis of the genotype of ES9-11, which was isolated from a 1,200-generation (gen) population of ES9, demonstrated distinct basal and salt stress response changes and genomic changes compared to the ancestor and strains allowed to evolve under nonstress conditions as controls (18, 19). As evolution of *D. vulgaris* Hildenborough reached 5,000 gen, we sought to explore the genomic and transcriptional changes and the changes in metabolites and PLFAs for a genotype isolated from 5,000 gen.

One mechanism of microbial adaptation to a stressful condition is a genetic change that confers restoration of cellular function to a prestressed state (15). Restoration of the global expression state or physiology back toward prestress levels has been observed in experimental evolution of *E. coli* under conditions with new carbon sources (20) or elevated temperature (21) and in evolution of genetically perturbed *E. coli* cells (22, 23). In these cases, stressors or genetic modifications affected gene expression and shifted the transcriptional and physiological states in the ancestors. The evolution time scales ranged from 250 gen to 2,000 gen. In the experimental evolution of *D. vulgaris* Hildenborough, mild salt stress stimulated very limited transcriptional changes in the ancestor. However, evolved *D. vulgaris* displayed a significantly altered transcriptional state under nonstress or transcriptional responses to high salinity (18). In this study, we aimed to explore the physiological, transcriptional, and metabolic states under nonstress and high-salinity conditions in a representative 5,000-gen genotype. Comparison between the representative genotypes at 5,000 gen and 1,200 gen, representing the best adaptation to salt stress among all tested clones, allowed us to determine whether a restorative mechanism was driving salt adaptation in *D. vulgaris* over a longer evolutionary time scale.

We hypothesized that changes of the key cellular components for salt tolerance, such as osmolytes and PLFAs, would remain as the number of generations increased but that most of the physiological and transcriptional changes in earlier generations would be restored. Whole-genome sequence analysis of a representative 5,000-gen genotype, ES10-5, revealed 36 newly acquired mutations affecting single genes, including three mutations located in the same genes as in the 1,200-gen representative genotype, ES9-11. Comparison between ES10-5 and ES9-11 demonstrated the persistent increase of basal abundance and responsiveness of glutamate and i17:1ω9c to high salinity, confirming their important roles in salt adaptation. Unique transcriptional responses to high salinity in ES10-5 included increased amino acid biosynthesis and transport, ion transport, and energy metabolism. The restorative trend of metabolic, physiological, and transcriptional changes in ES10-5 suggested that energy saving might be a major strategy for initial adaptation, and fine-tuned energy management was achieved in the long-term-adapted *D. vulgaris* Hildenborough.

## RESULTS

### Improved salt tolerance in 5,000-gen *D. vulgaris*.

We first evaluated the salt tolerance of six *D. vulgaris* populations (ES7 to ES12) that had been allowed to evolve for 5,000 gen under mild salt stress conditions. The growth phenotype was tested in a defined lactate-sulfate (LS4D) medium with 250 mM NaCl, a condition that led to an approximate 50% final biomass reduction in ancestral *D. vulgaris* Hildenborough (16, 17) and has been used as a high-salinity test condition (18, 19). The 5,000-gen ES populations grew to stationary phase within 48 h (see Fig. S1A in the supplemental material), in contrast to about 60 to 80 h for the 1,200-gen ES populations (18). Although similar final biomass yields (final optical density at 600 nm [OD_600_] ranged from 1.0 to 1.2) and average growth rates (~0.12 h^−1^) were observed in the 5,000-gen and 1,200-gen ES populations, the 5,000-gen ES populations had shorter lag phases and less variance among populations. The results suggested that salt adaptation involved a rapidly improved growth rate and growth efficiency and a gradual decrease in the lag phase time.

10.1128/mBio.01780-17.1 FIG S1  (A) Growth curves of 5,000-generation ES populations in LS4D (○) or LS4D plus 250 mM NaCl (●). (B) Growth curves of clones isolated from 5,000-generation ES10 population in LS4D (open symbols, dashed line), LS4D plus 300 mM NaCl (open symbols, solid line), or LS4D plus 400 mM NaCl (filled symbols, solid line). Download FIG S1, TIF file, 0.8 MB. Copyright © 2017 Zhou et al. 2017 Zhou et al. This content is distributed under the terms of the Creative Commons Attribution 4.0 International license.

**FIG 1:**
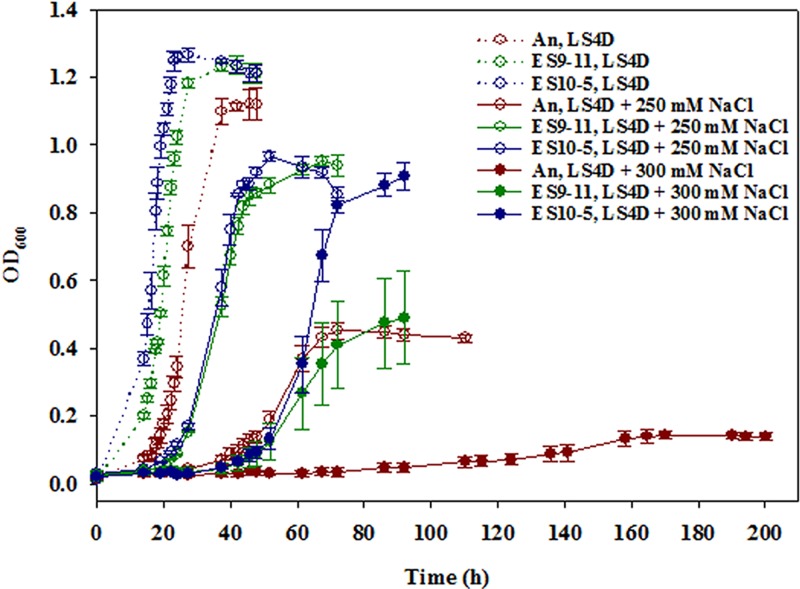
Growth curves of colony-based salt-evolved *D. vulgaris* Hildenborough strains ES10-5 and ES9-11 and the ancestral strain (An) grown in LS4D, LS4D plus 250 mM NaCl, or LS4D plus 300 mM NaCl.

Colony isolates were obtained by plating on a higher-salinity medium, LS4D plus 300 mM NaCl, since an extra 250 mM NaCl in LS4D did not represent a stringent condition to distinguish the variance among the 5,000-gen ES populations. Clones were obtained from ES10 only. A growth phenotype test of nine randomly selected clones demonstrated very similar growth in LS4D plus 300 mM NaCl. Variations among clones emerged when grown in LS4D plus 400 mM NaCl (Fig. S1B), a medium containing approximately 600 mM Na^+^ in total. No growth was observed in LS4D plus 500 mM NaCl. Due to its best growth performance with the shortest lag phase and the highest growth rate in LS4D plus 400 mM NaCl, ES10-5 was chosen as a representative 5,000-gen genotype representing the best adaptation to salt stress, and this strain was compared to the representative genotype ES9-11 strain, which was isolated from a 1,200-gen ES9 strain by similar means (18). Results of growth analysis of both clones (Fig. 1) were consistent with the growth test results for populations. In LS4D plus 250 mM NaCl, ES10-5 reached stationary phase about 12 h earlier than ES9-11, with similar final biomass yields; in LS4D plus 300 mM NaCl, ES10-5 had an approximate 2-fold-higher biomass yield than ES9-11. To better distinguish the differences between the two genotypes, LS4D plus 300 mM NaCl was used as a high-salinity condition for the subsequent metabolic, physiological, and transcriptional analyses.

### Genetic changes in ES10-5.

A total of 3,086,640 Illumina reads were obtained, and 36 mutations which affecte single genes (Table 1) were identified in ES10-5, with an average coverage of 132×. The mutations included 30 coding sequence mutations, 1 tRNA sequence mutation, and 5 intergenic region mutations. Among these, five mutations were derived from the preexisting polymorphisms and were present in both 1,200-gen ES9 and 1,200-gen ES10 (19). Most of the coding sequence mutations (25 out of 30; 83%) were nonsynonymous mutations which led to an amino acid change(s) that could potentially impact the function of the encoded protein. Among the 22 newly arising nonsynonymous mutations, 3 mutations occurred in genes DVU0597, DVU1204, and DVU2571 but at positions different from that of ES9-11, 3 mutations occurred in genes with unknown function, and 16 mutations occurred in genes potentially affecting amino acid transport, energy metabolism, or signal transduction. Occurrence of different alleles in independently evolved populations of ES10 and ES9 hinted at the importance of these genes in salt tolerance. In addition, a large deletion of 37,390 bp in the chromosome resulted in the deletion of 49 genes which encode prophage-related proteins or hypothetical proteins (Table S1). A large deletion of 105,758 bp in the plasmid resulted in about a 52% loss of plasmid DNA and the elimination of 80 genes (Table S2), including type III secretion system genes that are typically horizontally acquired. A 7,813-bp deletion in the chromosome was also found in ES9-11 (19), suggesting that genome reduction might be a common phenomenon in adaptive evolution.

10.1128/mBio.01780-17.5 TABLE S1  Genes affected by the large deletion of 37,390 bp (bp 233143 to 270532). Download TABLE S1, DOCX file, 0.02 MB. Copyright © 2017 Zhou et al. 2017 Zhou et al. This content is distributed under the terms of the Creative Commons Attribution 4.0 International license.

10.1128/mBio.01780-17.6 TABLE S2  Details of the genes deleted from the plasmid due to the deletion from bp 51979 to 157737. Download TABLE S2, DOCX file, 0.02 MB. Copyright © 2017 Zhou et al. 2017 Zhou et al. This content is distributed under the terms of the Creative Commons Attribution 4.0 International license.

10.1128/mBio.01780-17.7 TABLE S3  PCR primers used to confirm mutations with Sanger sequencing. Download TABLE S3, DOCX file, 0.02 MB. Copyright © 2017 Zhou et al. 2017 Zhou et al. This content is distributed under the terms of the Creative Commons Attribution 4.0 International license.

**TABLE 1:**
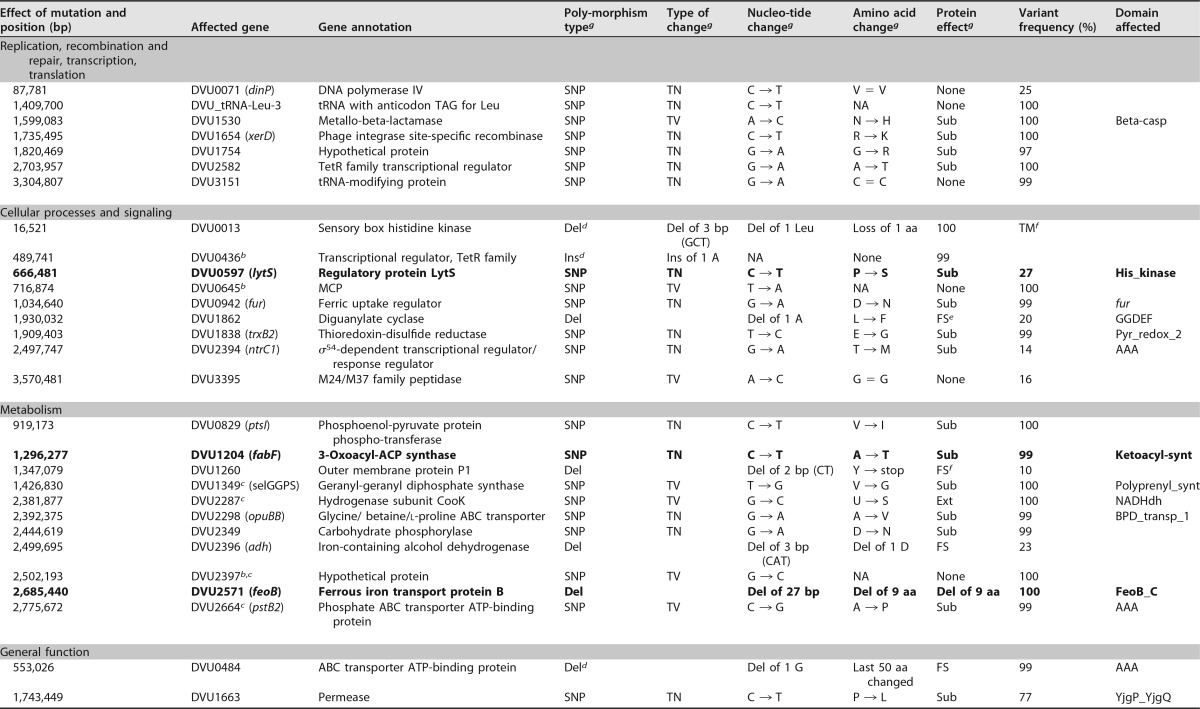

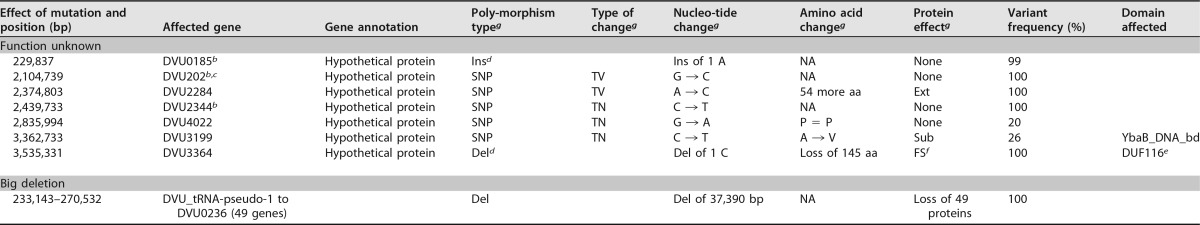
Mutations identified in strain ES10-5^a^

*^a^* Data shown in bold indicate that different mutation alleles were found in ES9-11.

*^b^* Mutation is located in the intergenic region.

*^c^* A preexisting polymorphism-derived mutation.

*^d^* The insertion or deletion affected the tandem repeat region.

*^e^* The domain was lost due to a frameshift mutation.

*^f^* A frameshift resulted in a premature stop codon.

*^g^* Abbreviations: Del, deletion; Ins, insertion; TN, transition; TV, transversion; Sub, substitution; FS, frameshift; Ext, extension; TM, transmembrane; NA, not applicable; aa, amino acid.

### Increased abundance of glutamate.

**FIG 2:**
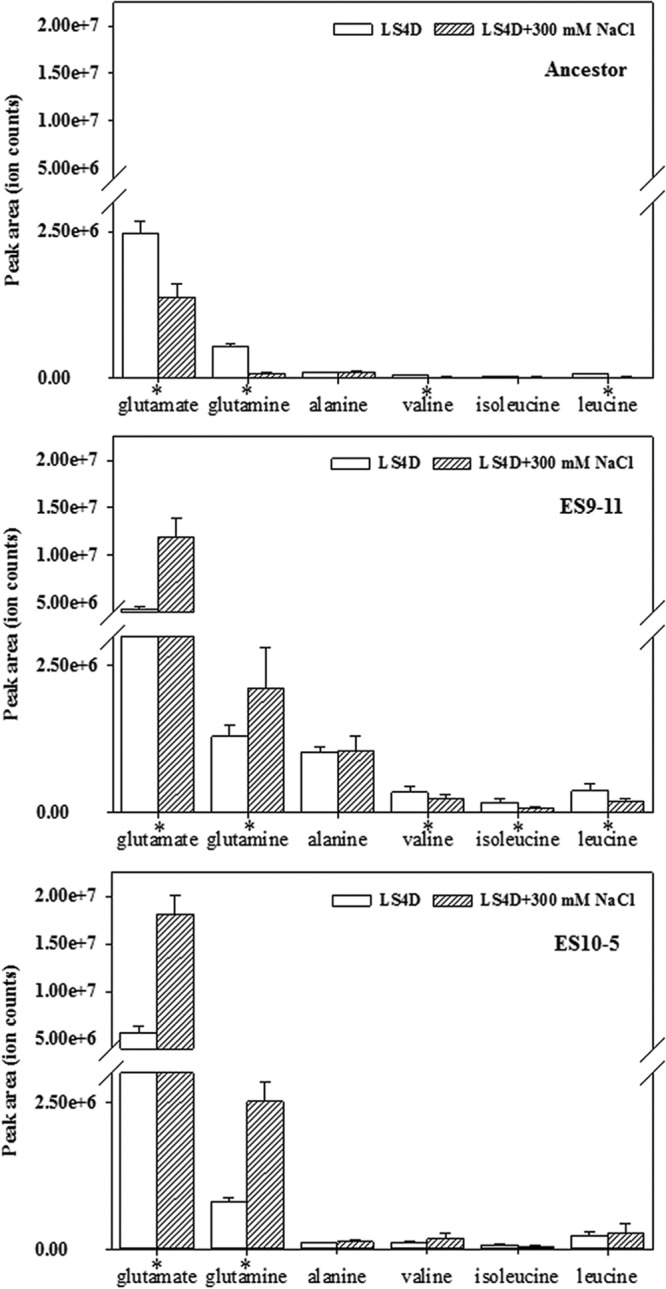
Accumulation of organic solutes in ES10-5, ES9-11, and the ancestral strain under nonstress conditions (LS4D) or high-salinity conditions (LS4D plus 300 mM NaCl). *, significant changes were induced under high-salinity conditions (LSD test, *P* < 0.05).

Intracellular accumulation of organic solutes is one of the major strategies to counteract external osmotic pressure. About 80 metabolites were detected by quadrupole time-of-flight (Q-TOF) analysis, and 13 were identified (Table S4). Among these, glutamate and glutamine were the two most abundant organic solutes in ancestor and evolved genotypes (Fig. 2). Under high salinity, different sets of metabolites showed abundance changes. In contrast to the overall decreased abundance of metabolites in the ancestor strain, we observed increased abundance of glutamate and glutamine and decreased abundance of valine, isoleucine, and leucine in ES9-11, and in ES10-5 we observed an increased abundance of glutamate and glutamine. Increased basal abundance of glutamate and abundance under high salinity in both ES10-5 and ES9-11 confirmed the key role of glutamate in salt adaptation. Alanine was not responsive to high salinity in any of the strains. Abundance levels of alanine, valine, and isoleucine were higher in ES9-11, while similar abundances were observed in ES10-5 and the ancestor under nonstress and high-salinity conditions. Alanine and these branched amino acids were effective in alleviating salt stress in a growth experiment (17). Branched amino acids could be potential ammonium donors in biosynthesis of glutamate (24). Our results suggested that alanine and branched amino acids might be important for early stages of salt adaptation.

10.1128/mBio.01780-17.8 TABLE S4  Metabolites detected by Q-TOF. Download TABLE S4, DOCX file, 0.04 MB. Copyright © 2017 Zhou et al. 2017 Zhou et al. This content is distributed under the terms of the Creative Commons Attribution 4.0 International license.

### Abundance changes of PLFAs.

**FIG 3:**
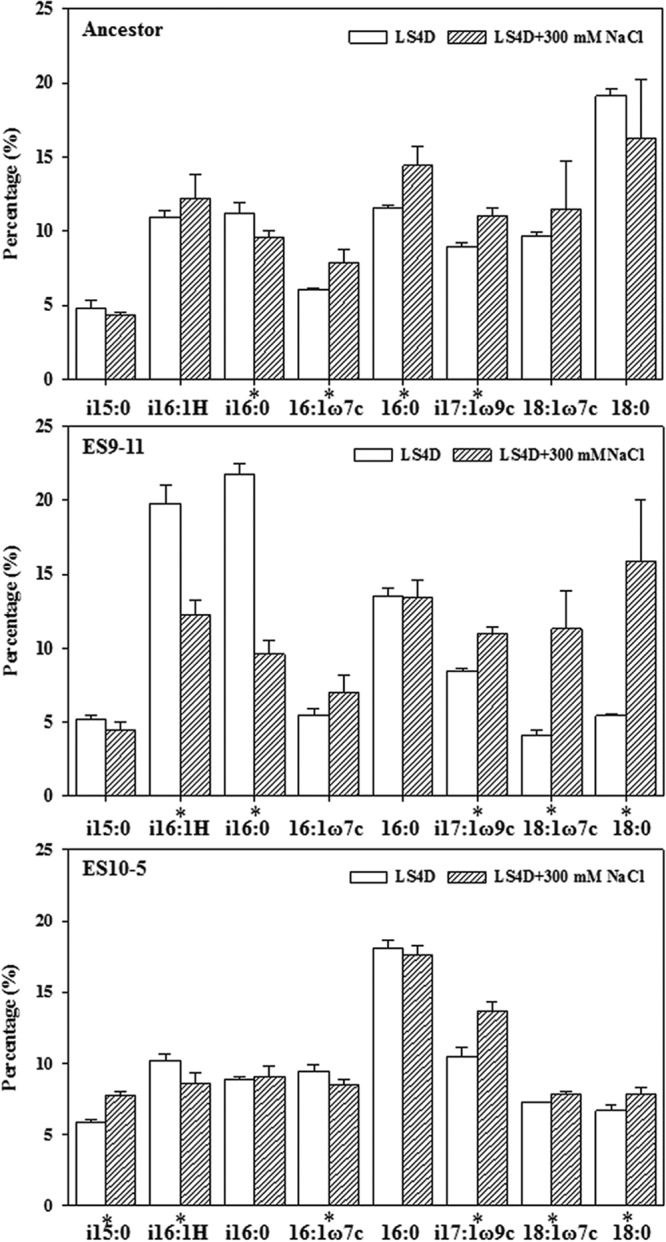
PLFA compositions in ES10-5, ES9-11, and the ancestral strain under nonstress conditions (LS4D) or high-salinity conditions (LS4D plus 300 mM NaCl). *, significant change induced by high salinity (LSD test, *P* < 0.05).

Membrane PLFA composition changes, such as increased percentages of unsaturated PLFAs or branched PLFAs, compensate for decreases in membrane fluidity under high salinity. Analysis of the relatively abundant (>5%) PLFAs demonstrated that different sets of PLFAs displayed abundance changes under high-salinity conditions in the ancestor or the evolved strains (Fig. 3). An increase of branched PLFA i17:1ω9c was common in all strains, while an increase of i15:0 was unique to ES10-5. The relative abundances of these two branched PLFAs were the highest in ES10-5 under nonstress and high salinity, indicating their important roles in salt tolerance. Relative abundances of the other branched PLFAs, such as i16:0, 16:1ω7c, and 18:1ω7c, were similar in all strains under high salinity, although their basal abundances were quite different under nonstress conditions (highest percentages were observed for 18:1ω7c in the ancestor, i16:0 in ES9-11, and 16:1ω7c in ES10-5) (least significant difference [LSD] test, *P* < 0.05).

A higher unsaturation index (UI) and increased percentages of branched PLFAs indicate a more fluid membrane. Under nonstress conditions, ES10-5 had the highest UI and ES9-11 had the highest percentage of branched PLFAs. Under high salinity, all strains had similar UIs, but ES10-5 had the highest percentage of branched PLFAs (LSD test, *P* < 0.05) (Fig. S2). These results confirmed the importance of both unsaturated PLFAs and branched PLFAs for salt tolerance, but branched PLFAs might play a more important role.

10.1128/mBio.01780-17.2 FIG S2  Unsaturation index (A) and percent branched PLFAs (B) in strains ES10-5 and ES9-11 and the ancestral strain (An) under nonstress (LS4D) or high-salinity (LS4D plus 300 mM NaCl) conditions. *, significant change induced by high salinity (*P* < 0.05). Download FIG S2, TIF file, 0.7 MB. Copyright © 2017 Zhou et al. 2017 Zhou et al. This content is distributed under the terms of the Creative Commons Attribution 4.0 International license.

### Increased cell motility in ES10-5.

**FIG 4:**
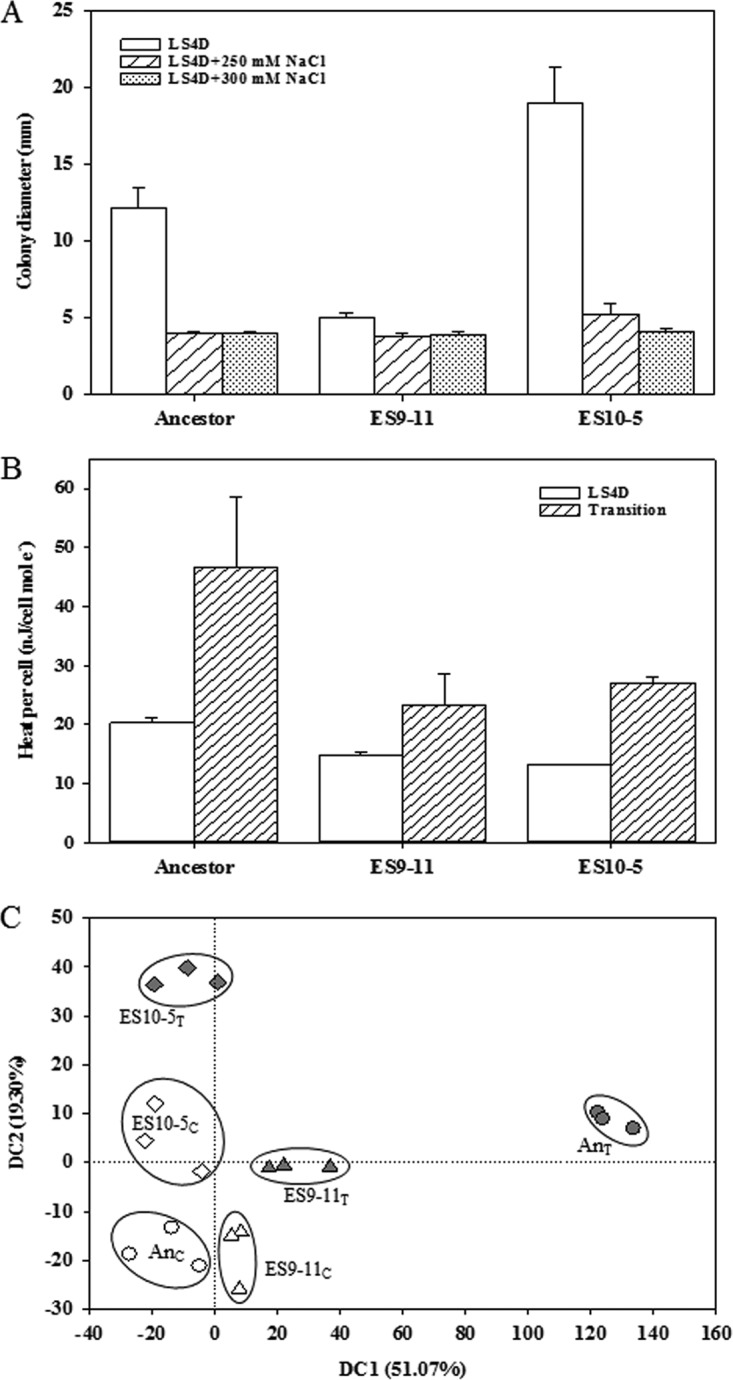
Physiological and transcriptional responses to high salinity for ES10-5, ES9-11, and the ancestral strain (An). (A) Cell motility. (B) Heat generated per cell under nonstress conditions (LS4D) and after transfer into high-salinity medium (LS4D plus 250 mM NaCl; transition). (C) DCA results for global transcriptional profiles. A subscript C after a strain designation indicates cells were cultured in LS4D; a subscript T after a strain designation indicates cells were cultured in LS4D plus 300 mM NaCl.

Decreased cell motility was observed for ES9-11 (18). To determine whether the same trend remained in ES10-5, the cell motility of ES10-5 was examined in LS4D medium supplemented with 250 or 300 mM NaCl (Fig. 4A; Fig. S3). High salinity inhibited cell motility in all strains. In both LS4D and LS4D plus 250 mM NaCl, ES9-11 showed decreased cell motility compared to the ancestor, which was consistent with previous results (18). However, ES10-5 had the highest cell motility (*t* test, *P* < 0.01). An additional 300 mM NaCl in the medium almost completely arrested cell motility. However, ES10-5 showed the best growth, as indicated by the dark color of the colonies generally seen with *D. vulgaris*. The observed higher cell motility of ES10-5 might be an indicator of better energy management, as cell motility is a process with a high energy cost.

10.1128/mBio.01780-17.3 FIG S3  Cell motilities of the ancestral strain and the evolved strains ES10-5 and ES9-11 in LS4D (A), LS4D plus 250 mM NaCl (B), and LS4D plus 300 mM NaCl (C). Download FIG S3, TIF file, 1 MB. Copyright © 2017 Zhou et al. 2017 Zhou et al. This content is distributed under the terms of the Creative Commons Attribution 4.0 International license.

### Improved growth energy efficiency.

To assess overall growth energy efficiency, the total energy usage in each strain was measured by tracking dissipated heat via microcalorimetry, which employs the concept of describing the energy expense of microbial growth in terms of increased enthalpy (25). In LS4D medium, the heat released per cell was the lowest in ES10-5, intermediate in ES9-11, and the highest in the ancestor. After transferring to LS4D plus 250 mM NaCl, the heat released per cell was similar in ES10-5 and ES9-11 and much higher in the ancestor (Fig. 4B). The growth energy efficiencies in LS4D plus 300 mM NaCl were not measured, due to the slow growth. These data indicated that the improvement of growth energy efficiency in evolved strains might provide an advantage for selection during evolution.

### Overall transcriptional profile changes.

To determine the transcriptional changes associated with the physiological changes and improved salt tolerance, the transcriptional profile of ES10-5 was examined and compared with those of ES9-11 and the ancestor. Under nonstress conditions, the numbers of genes with significantly increased or decreased expression, respectively, compared to the ancestor were 52 and 123 in ES10-5 and 62 and 88 in ES9-11. By eliminating the genes affected by deletion (95 genes in ES10-5 and 8 genes in ES9-11 [Table S6]), the actual number of genes with significant expression changes was lower in ES10-5. High salinity induced the smallest transcriptional changes in ES10-5, an intermediate number of changes in ES9-11, and the largest number of changes in the ancestor (Fig. 4C). The numbers of genes with significantly increased or decreased expression were 71 and 128 in ES10-5 and 36 and 586 in ES9-11, and almost all the genes in the ancestor showed decreased expression. The microarray data were validated by quantitative reverse transcription-PCR (qRT-PCR) analysis of 36 genes which were differentially expressed in the ancestor and evolved strains (Pearson coefficient *r* = 0.86, *P* < 0.001) (Fig. S4). Large-scale decreased gene expression under high salinity in the ancestor might have been due to poor growth. Here, we focused on the basal transcriptional changes under nonstress conditions and transcriptional changes under high salinity to distinguish (i) the common changes in ES10-5 and ES9-11, (ii) the novel changes in ES10-5, and (iii) the changes that occurred only in ES9-11. The first two categories might represent the adaptive changes, and the third category might be important for early-stage adaptation. We have reported here genes in the format of operons which showed the same trend of expression changes with at least one gene in the multigene operon showing more than a 3-fold change.

10.1128/mBio.01780-17.4 FIG S4  (A) Correlation between microarray data and qRT-PCR data for selected genes in strains ES10-5 and ES9-11 compared to those for the ancestor (An) strain in LS4D. (B) Gene expression changes with growth in LS4D plus 300 mM NaCl compared to LS4D medium in all strains. Operons are separated by dot lines. Download FIG S4, TIF file, 0.6 MB. Copyright © 2017 Zhou et al. 2017 Zhou et al. This content is distributed under the terms of the Creative Commons Attribution 4.0 International license.

10.1128/mBio.01780-17.9 TABLE S5  Selected genes and gene-specific primers for real-time RT-PCR. Download TABLE S5, DOCX file, 0.02 MB. Copyright © 2017 Zhou et al. 2017 Zhou et al. This content is distributed under the terms of the Creative Commons Attribution 4.0 International license.

10.1128/mBio.01780-17.10 TABLE S6  Expression of genes affected by large deletions in chromosome or plasmid DNA in LS4D. Download TABLE S6, DOCX file, 0.03 MB. Copyright © 2017 Zhou et al. 2017 Zhou et al. This content is distributed under the terms of the Creative Commons Attribution 4.0 International license.

### Basal transcriptional changes.

Under nonstress conditions (Table 2), both ES10-5 and ES9-11 had increased expression of genes involved in glutamate and tryptophan synthesis, iron uptake, and genetic material exchange, such as pilin genes. ES10-5 had increased expression of acetolactate genes (ALS), which are involved in branched amino acid biosynthesis (26), and *ech* hydrogenase genes. For cell motility-related genes, ES9-11 had decreased expression of flagellar biosynthesis genes and increased expression of four methyl-accepting chemotaxis protein (MCP) genes. ES10-5 had increased expression of one MCP gene, DVU0094. Increased expression of genes involved in ion transport (e.g., operons DVU2378 to -2390, DVU0100 and -0101, and DVU0102 to -0104), energy metabolism (e.g., periplasmic [Ni-Fe] hydrogenase genes), and transcriptional regulator genes were only observed in ES9-11. These transcriptional changes might be important for early-stage adaptations.

**TABLE 2:**
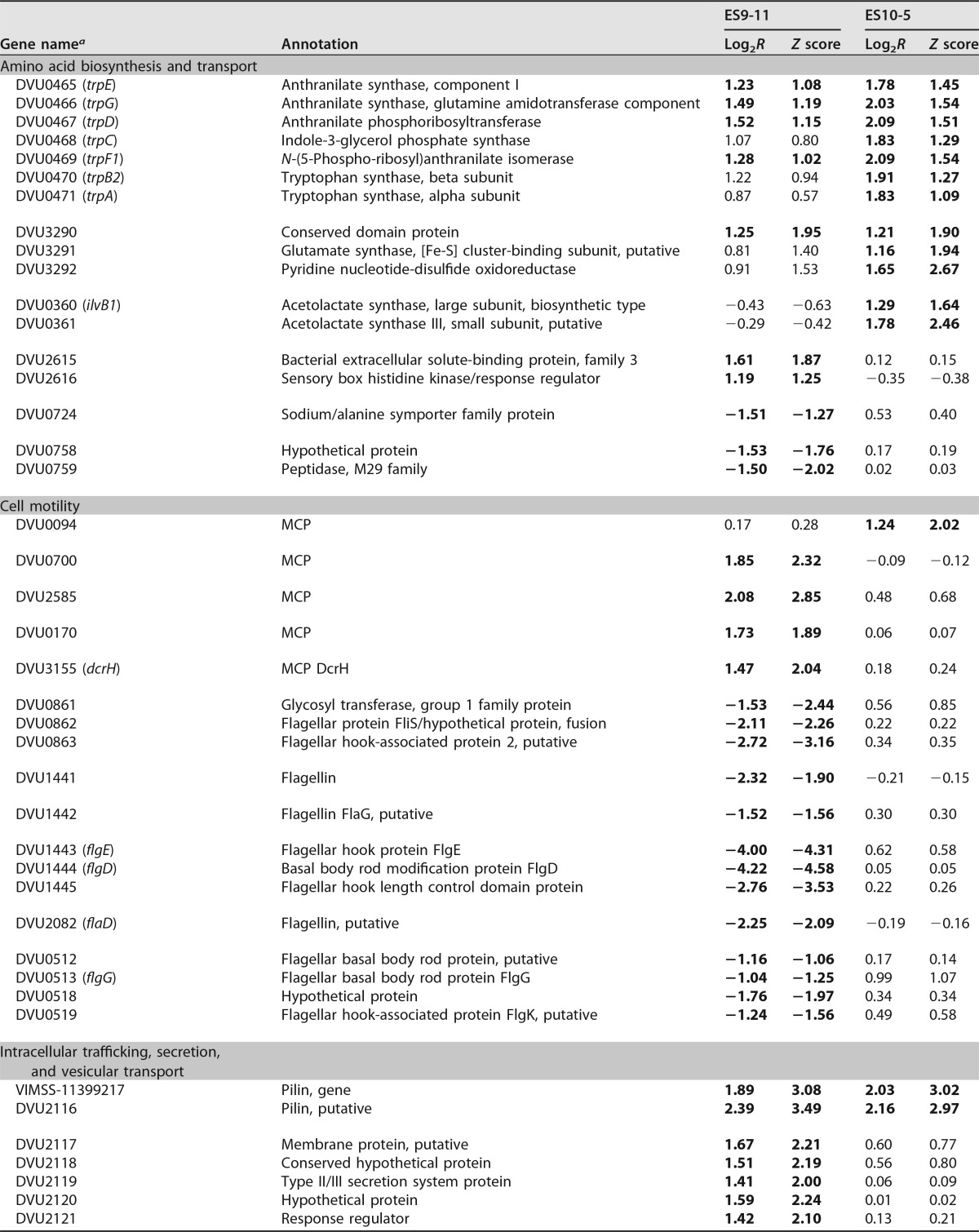

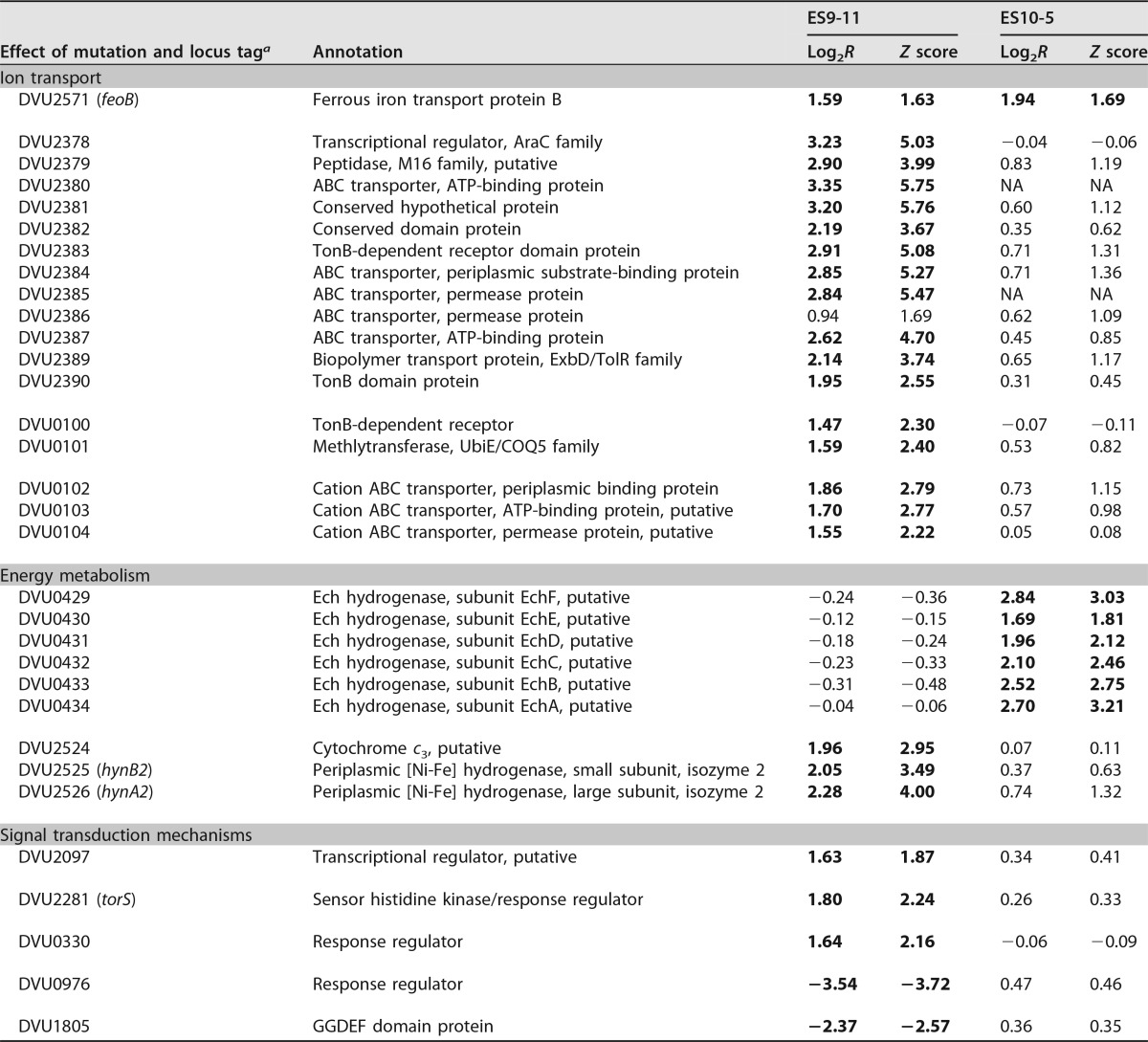
Basal gene expression changes relative to expression by the ancestor under nonstress conditions

*^a^* Operons are grouped together. Expression levels that increased (positive values) or decreased (negative values) more than 2-fold are indicated in boldface. NA, no data available.

### Transcriptional changes under high salinity.

Gene expression changes in the following gene categories were examined (our findings are summarized in Table 3).

**TABLE 3:**
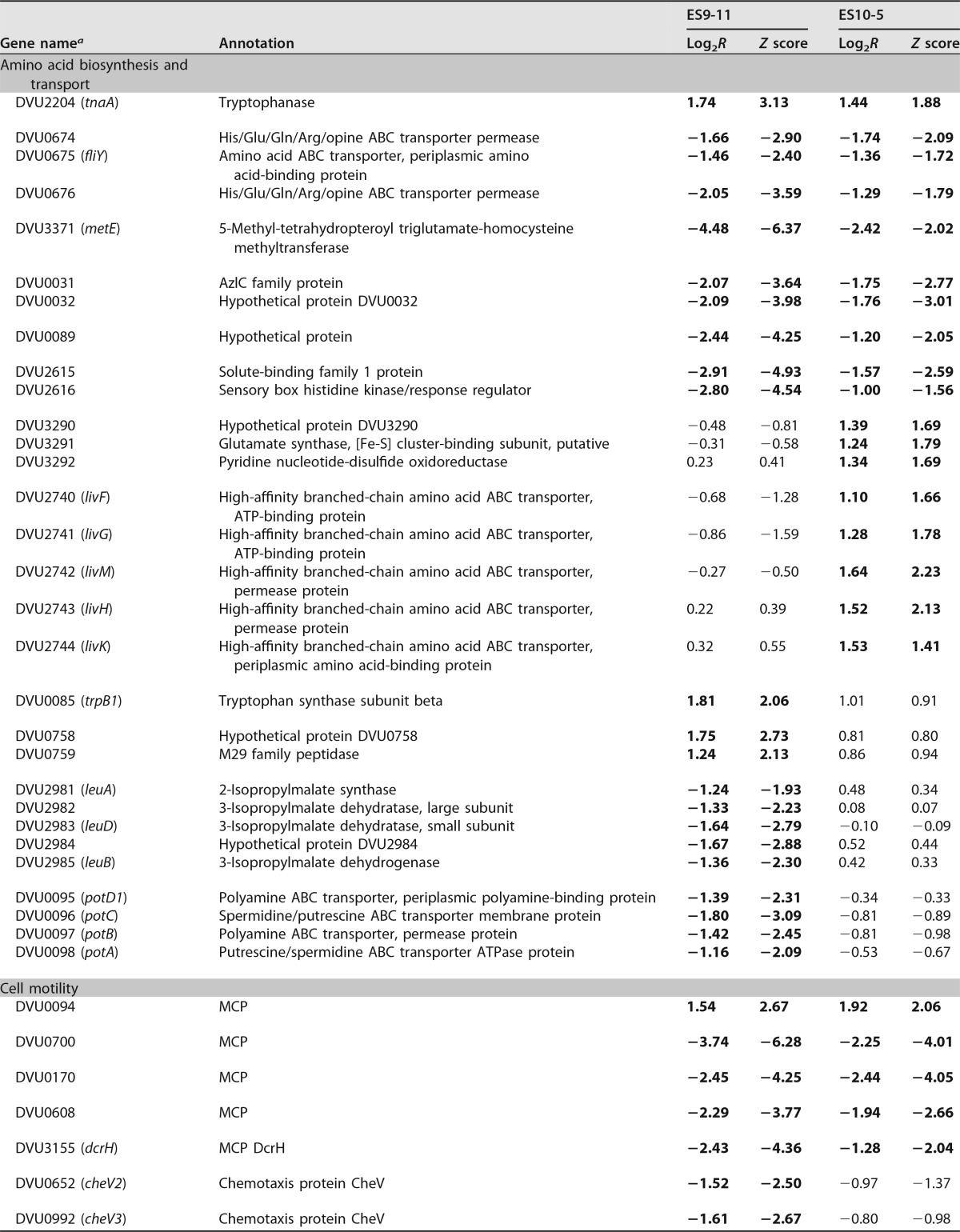

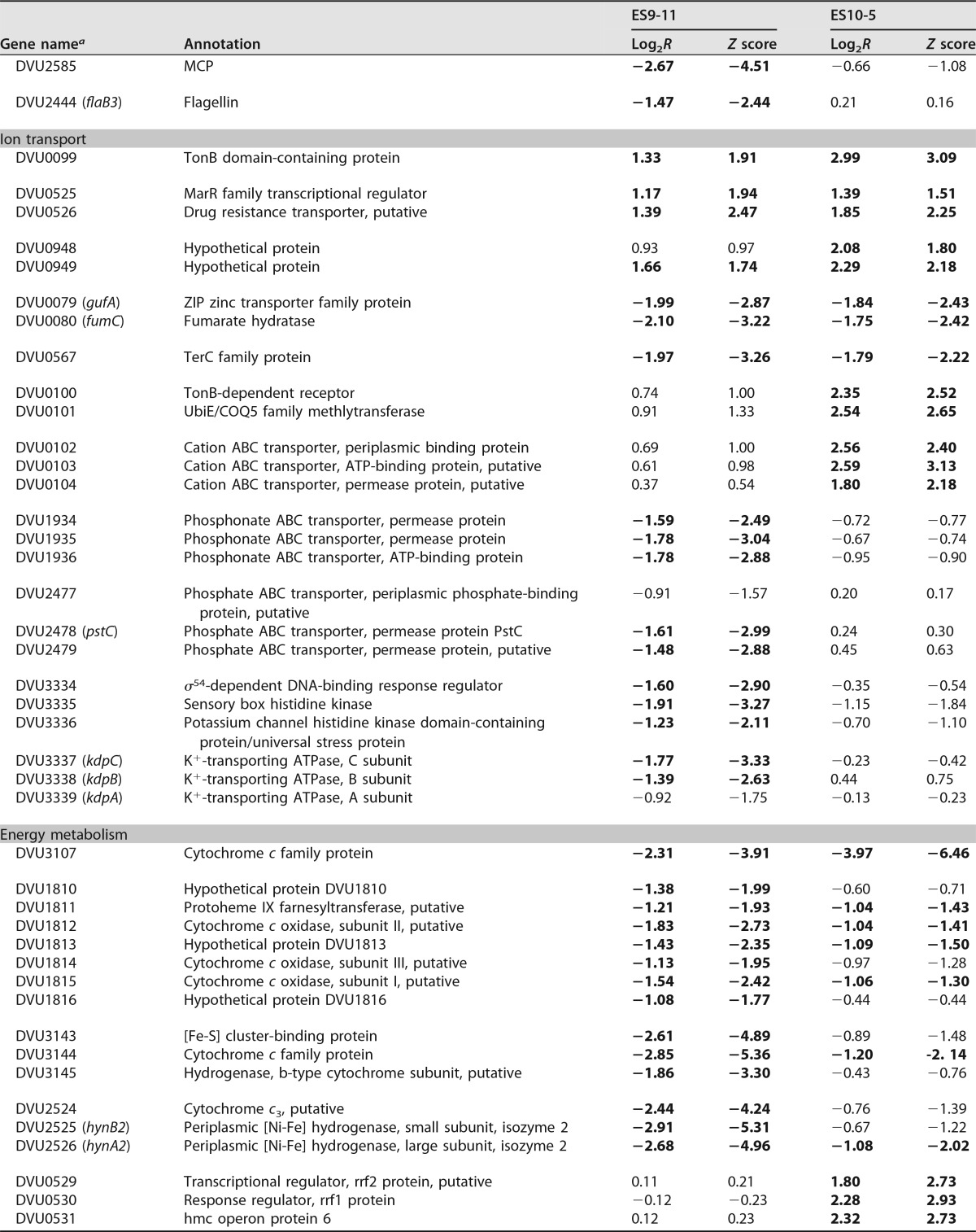

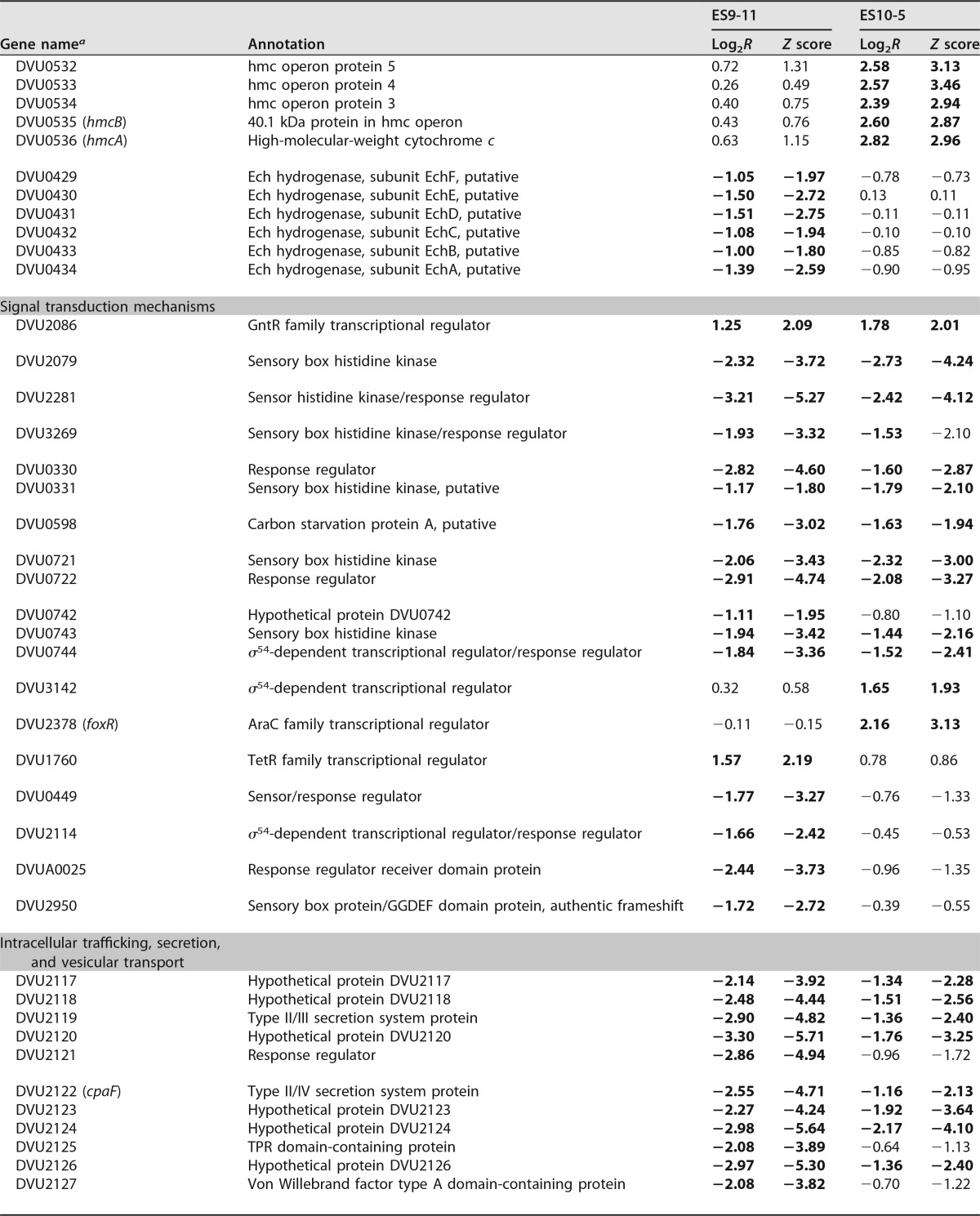

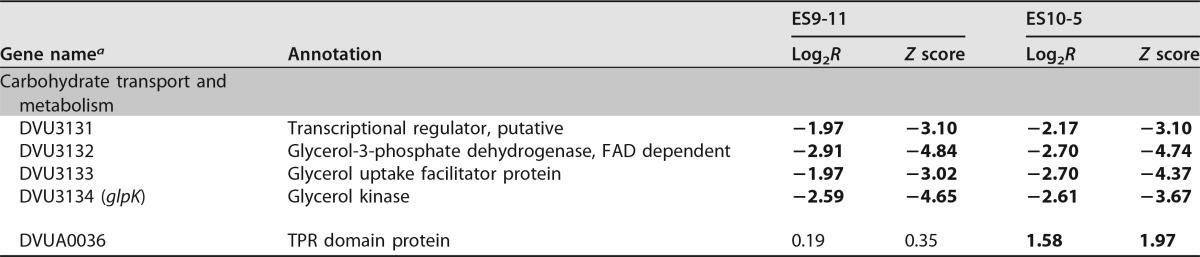
Gene expression changes stimulated by high salinity

*^a^* Operons are grouped together. Expression levels that increased (positive values) or decreased (negative values) more than 2-fold are indicated in boldface.

### (i) Amino acid synthesis, transport, and metabolism.

Both ES10-5 and ES9-11 had increased expression of tryptophan synthesis genes and decreased expression of methionine biosynthesis genes, solute-binding protein genes, and genes involved in transport of glutamate or branched amino acids. ES10-5 had increased expression of glutamate biosynthesis genes and branched amino acid transport genes. ES9-11 had decreased expression of leucine biosynthesis genes. The results were consistent with the increased abundance of glutamate in ES10-5 and the decreased abundance of leucine in ES9-11. In addition, ES9-11 had decreased expression of polyamine transport genes, which have been shown to be important for stress tolerance in plants (27).

### (ii) Cell motility.

Both ES10-5 and ES9-11 had increased expression of MCP gene DVU0094 and decreased expression of three MCP genes. ES9-11 had decreased expression of three additional MCP genes and a flagellum biosynthesis gene, *flaB3* (DVU2444). The results suggested that both MCP and flagellum biosynthesis genes might contribute to the cell motility decrease under salt stress but that decreased flagellum biosynthesis is important for early-stage adaptation.

### (iii) Ion transport.

Both ES10-5 and ES9-11 had increased expression of a TonB domain-containing protein gene, DVU0099, that is potentially involved in uptake of cobalamin and iron, as DVU0949 contains an ABC-type Co^2+^ transport domain and drug efflux pump genes DVU0525 to -0526 and shows decreased expression of DVU0079, which is potentially involved in zinc transport, and DVU0567, which is potentially involved in efflux of tellurium ions. In addition, ES10-5 had increased expression of a TonB-dependent receptor gene, DVU0100, and cation ABC transporter genes DVU0102 to -0104; higher basal expression of these genes was also found in ES9-11, suggesting the importance of these genes at both early and later stages during adaptation. Decreased expression of genes involved in transport of phosphate, phosphonate, or potassium was observed only in ES9-11.

### (iv) Energy metabolism.

Both ES10-5 and ES9-11 had decreased expression of cytochrome *c* genes and periplasmic [Ni-Fe] hydrogenase genes. ES10-5 had increased expression of *hmc* genes, while ES9-11 had decreased expression of *ech* genes. As ES10-5 had higher basal expression of *ech* genes, the results suggested that ES10-5 had an overall higher expression of energy metabolism genes. Expression of [Ni-Fe] hydrogenase genes DVU2525 and -2526 increased under nonstress conditions but decreased under high-salinity conditions in ES9-11, suggesting that expression of these genes might be unfavorable for overall energy efficiency.

### (v) Signal transduction mechanisms.

Both ES10-5 and ES9-11 had increased expression of a GntR family transcriptional regulator gene, DVU2086, and decreased expression of the two-component system genes DVU0330 and -0331, DVU0721 and -0722, and DVU0743 and -0744. In addition, ES10-5 had increased expression of the sigma-54-dependent transcriptional regulator gene DVU3142 and an AraC family transcriptional regulator gene, DVU2378. AraC and GntR family transcriptional regulators have been shown to be involved in stress responses in other microorganisms (28, 29). ES9-11 had increased expression of the TetR family transcriptional regulator gene DVU1760 and additional response regulators, such as DVU2114, which is involved in regulating pili (30).

### (vi) Genes involved in other cellular pathways.

Both ES10-5 and ES9-11 had decreased expression of genes involved in carbohydrate transport and metabolism or in intracellular trafficking, secretion, and vesicular transport. Genes in the latter category are involved in the salt stress response of other organisms (31, 32). Increased expression of general stress response genes, such as phage shock protein genes DVU2986 and DVU2988 and heat shock protein genes DVU2441 and DVU2442, was only observed in ES9-11 (Table S7). Expression of general stress response genes was also found after short-term adaptation to salt stress in the ancestral *D. vulgaris* Hildenborough (17), suggesting that the general stress response is involved in early-stage adaptation.

10.1128/mBio.01780-17.11 TABLE S7  High-salinity-induced gene expression changes. Download TABLE S7, DOCX file, 0.02 MB. Copyright © 2017 Zhou et al. 2017 Zhou et al. This content is distributed under the terms of the Creative Commons Attribution 4.0 International license.

## DISCUSSION

Rapid phenotypic and genetic adaptations are common in experimental evolution (1, 33). The aim of this study was to identify the key cellular components for salt adaptation in *D. vulgaris* Hildenborough and reveal the mechanistic changes associated with improved salt tolerance over an evolutionary time scale. Two genotypes, ES10-5 and ES9-11, representing the best salt adaptation phenotypes at 5,000 gen and 1,200 gen, respectively, were analyzed. Given the differences in the duration of evolution and salt tolerance phenotype and the presence of common mutations in ES10-5 and ES9-11, comparison of the two genotypes shed light on the mechanistic changes of salt adaptation over time. The results indicated the key roles of glutamate and the branched PLFA i17:1ω9c for salt tolerance in *D. vulgaris* Hildenborough.

The overall basal physiological and transcriptional changes and responses to high salinity in ES10-5 were less dramatic than those in ES9-11. First, the basal abundance levels of most organic solutes in ES10-5 were similar to those of the ancestor, and glutamate and glutamine were the only two metabolites that were responsive to high salinity in ES10-5 (Fig. 2). Second, the magnitudes of basal and responsive changes (relative percentages) of PLFA were smaller in ES10-5 (Fig. 3). Third, the numbers of genes with significant expression changes were lower in ES10-5. Basal transcriptional changes, potentially important for the initial adaptation, have been observed also in experimentally evolved yeast (*Saccharomyces cerevisiae*) (34). Diminished basal physiological and transcriptional changes in ES10-5 might be indicative of adaptation via optimization of transcriptional and physiological responses to salt stress, similar to the observations in evolution of metabolically perturbed or stress-perturbed microorganisms (15, 20–23).

The mechanistic differences between ES10-5 and ES9-11 suggested that growth energy efficiency might be a key factor for selection during salt adaptation. Under nonstress conditions, decreased expression of flagellum synthesis genes and increased ion transport genes were only observed in ES9-11. Under high-salinity conditions, ES9-11 had decreased expression of flagellar synthesis genes and phosphate or potassium transport genes. ES10-5 had increased expression of genes involved in energy metabolism, glutamate biosynthesis, and ion transport. Glutamate biosynthesis and flagellar biosynthesis are processes with high energy costs (35). Decreased expression of flagellum biosynthesis genes and decreased cell motility in ES9-11 might be indicative of an energy-saving strategy. Decreased expression of flagellar biosynthesis genes was also observed in the acute response of *D. vulgaris* to salt shock (16) and of high-temperature-evolved *E. coli* (21), suggesting that decreased cell motility might be a common immediate strategy under stress conditions. Higher expression of the energy metabolism genes *ech* and *hmc* in ES10-5 (Table 2 and 3) might help to meet the increased energy demand. In addition, genome reduction, resulting from large deletions in the chromosome (7.8 kb from ES9-11 and 37.4 kb from ES10-5) and the plasmid DNA (106 kb from ES10-5) might contribute to better energy efficiency management (36). We speculate that energy-efficient individuals were selected during evolution, possibly due to faster growth.

The results also demonstrated the complex relationship between a genotype and a phenotype. ES10-5 had more mutations than ES9-11 (36 versus 10 mutations affecting single genes). The two genotypes shared five preexisting polymorphism-derived mutations and different alleles in three genes. Surprisingly, their growth performances were similar, except that ES10-5 entered the stationary growth phase about 12 h earlier when grown in LS4D plus 250 mM NaCl. The superior salt tolerance of ES10-5 became obvious when tested in LS4D plus 300 mM NaCl (Fig. 1). The results confirmed the large contribution of the early or first-step mutations in adaptation, as observed in evolution of other microorganisms (1, 33). Despite new mutations in genes that potentially affect salt tolerance in ES10-5, such as ABC transporters, permeases, and membrane proteins (Table 1), it is challenging to link these mutations to physiological changes and salt tolerance, possibly due to the negative epistasis that occurs among beneficial mutations (37, 38).

In conclusion, comparison of *D. vulgaris* strains allowed to evolve for a different number of generations revealed glutamate as the key osmolyte and i17:1ω9c as a key PLFA for salt tolerance and the mechanistic changes associated with improved salt tolerance. Future studies will focus on analysis of gene function and gene regulatory networks in multiple populations to dissect the molecular mechanisms of salt adaptation.

## MATERIALS AND METHODS

### Bacterial strains and growth conditions.

Six replicate *Desulfovibrio vulgaris* Hildenborough populations (salt stress evolved, strains ES7 to -12) were allowed to experimentally evolve in defined LS4D medium (60 mM lactate as the electron donor and 50 mM sulfate as the electron acceptor) supplemented with 100 mM NaCl (18) for 5,000 gen. Populations were archived at 100-gen intervals as glycerol stocks (1-ml stationary-phase cultures mixed with 0.5 ml of 50% glycerol) and stored at −80°C. Single colonies were obtained by plating on LS4D with 300 mM NaCl (1% agar). Randomly picked clones were propagated in liquid LS4D plus 300 mM NaCl for 3 days before transferring to LS4D (10% inoculum) for one more round of growth (48 h) to reduce the NaCl carryover in glycerol stocks. Strain ES9-11 was isolated from the 1,200-gen population ES9 (18).

For subsequent experiments, glycerol stocks (150 µl) were revived in LS4D (10 ml). After 48 h of growth, a 1% inoculum was transferred into production vessels or Hungate tubes for biomass production or growth phenotype tests.

### Whole-genome sequencing.

Genomic DNA was isolated from ES10-5 by using a modified cetyltrimethylammonium bromide method (39). The sequencing library was prepared with the Nextera DNA sample preparation kit (catalog number FC-121-1031; Illumina, San Diego, CA) and sequenced with a MiSeq benchtop sequencer (Illumina) in a 2× 250-cycle format. Alignments to the *D. vulgaris* Hildenborough reference sequences (NC_002937.3 [chromosome] and NC_005863.1 [plasmid]) and mutation calls (single nucleotide polymorphisms [SNP], insertions, or deletions) were performed with Geneious 9.1.5 (Biomatters Ltd.). Final calls of mutations were manually checked and confirmed by PCR and Sanger sequencing (Table S3).

### Metabolites assay.

Metabolites were extracted from mid-exponential-phase culture pellets (five replicates) with a cold methanol method (40) and analyzed using an Agilent 6550 Q-TOF apparatus (Agilent Technologies, Santa Clara, CA). Liquid chromatography (LC) separation was conducted on an Agilent 1290 ultrahigh-performance LC (UHPLC) system (Agilent Technologies, Santa Clara, CA) with a 150- by 2.1-mm, 5-µm, 200-Å SeQuant ZIC-pHILIC column (EMD Millipore, Billerica, MA) and guard column of 20 by 2.1 mm, 5 µm (EMD Millipore, Billerica, MA) (41). MassHunter (Agilent) was used for initial data analysis, and Metabolite Atlas was used for targeted data analysis. Compounds were confirmed using authentic standards.

### PLFA and motility assays.

Late-exponential-phase cultures (four replicates) were harvested for PLFA assays (Microbial ID Inc., Newark, DE) (18). Briefly, fatty acids were extracted, methylated, and then analyzed on a gas chromatograph equipped with a flame ionization detector. Peaks were identified with Sherlock software (Microbial ID Inc.).

To test cell motility, mid-exponential-phase cell cultures (5 µl, OD_600_ of ~0.4) were applied to the surfaces of 0.4% (wt/vol) soft agar LS4D plates supplemented with 0, 250 mM, or 300 mM NaCl. After 4 days at 37°C, the colony diameters were measured.

### Microcalorimetric assay.

The microcalorimetric measurements were performed on a TAM III nanocalorimeter (TA Instruments, New Castle, DE), which measures the heat flow between a reaction cell and reference cell (42–44). Prior to each experiment, the heat flow response of the calorimeter was calibrated by electrical heating of 4 ml Hastelloy in the reaction cell and 3 ml of LS4D or LS4D plus 250 mM NaCl in the reference cell at 37°C, and the response was verified by measuring the heat of protonation of Tris-hydroxymethylaminomethane (Tris-THAM) at 25°C (45). After calibration, the precultures from LSD medium were inoculated (10%) into Balch tubes, and 3 ml of culture was immediately dispensed into a sterile reaction cell in an anoxic glove box and transferred into the calibrated microcalorimeter. After the heat flow reached baseline, the reaction cell was removed and sampled for cell counts with Sybr green (46, 47). Heat levels during growth were derived by integration of the heat flow curves. The total heat was normalized to the total cell number in a vial.

### Transcriptomics and real-time qRT-PCR analysis.

Triplicate cultures grown in LS4D (OD_600_, ~0.4) or LS4D plus 300 mM NaCl (OD_600_ values of ~0.4 for ES10-5, ~0.3 for ES9-11, and ~0.15 for the ancestor) were harvested by spinning 50-ml cell cultures at 6,000 × *g* for 10 min at 4°C. The pellets were frozen in liquid nitrogen and stored at −80°C.

Total RNA was isolated with the NucleoSpin RNA II system (catalog number 740955; Clontech). Labeling of total RNA (2 µg per sample) with Cy3 and of genomic DNA (gDNA; 1.5 µg per labeling reaction mixture) with Cy5 was conducted as described previously (48). Cy3-cDNA and Cy5-gDNA (1/12 of one labeling reaction product) were cohybridized to one array on custom 12-plex NimbleGen oligonucleotide microarrays for 16 h at 42°C. The microarray consisted of 9,619 gene-specific 65-mer probes (three probes per gene and six replicates per probe), negative controls (1,689 probes specific to thermophiles), and positive controls (1,200 probes specific to 16S). After washing and drying, the arrays were scanned with a NimbleGen MS200 microarray scanner (Roche NimbleGen, Madison, WI) at 100% laser power and 100% PMT (photomultiplier tube) level. The transcript abundance was calculated as the ratio of the signal intensities with Cy3 and Cy5. Gene expression changes with |*Z*|scores of ≥1.5 and |log_2_*R*|values of ≥1.5 were considered significant. Genes detected in at least two out of three biological replicates were kept for detrended correspondence analysis (DCA). qRT-PCR of 36 genes (Table S4) was conducted (17) to validate the microarray data.

### Accession number(s).

The microarray data were deposited in the NCBI GEO database under accession number GSE103797.
