# Supplementary figures and images for: Key Metabolites and Mechanistic Changes for Salt Tolerance in an Experimentally Evolved Sulfate-Reducing Bacterium, Desulfovibrio vulgaris
Source: mBio. 2017 Nov 14;8(6):e01780-17. doi: 10.1128/mBio.01780-17 (PMC5686539; doi:10.1128/mBio.01780-17)

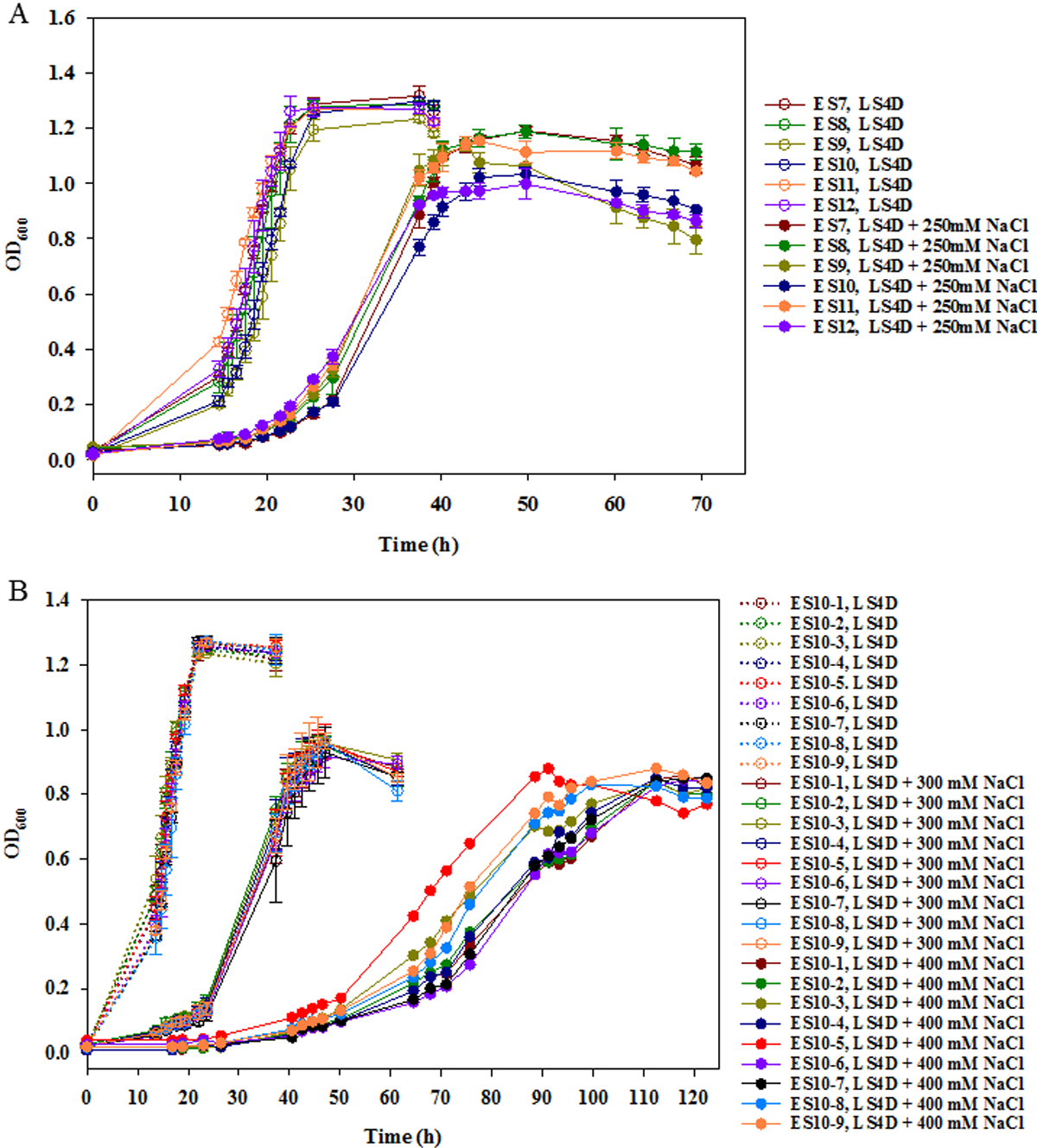

Supplement: FIG S1 [file mbo006173579sf1.tif]

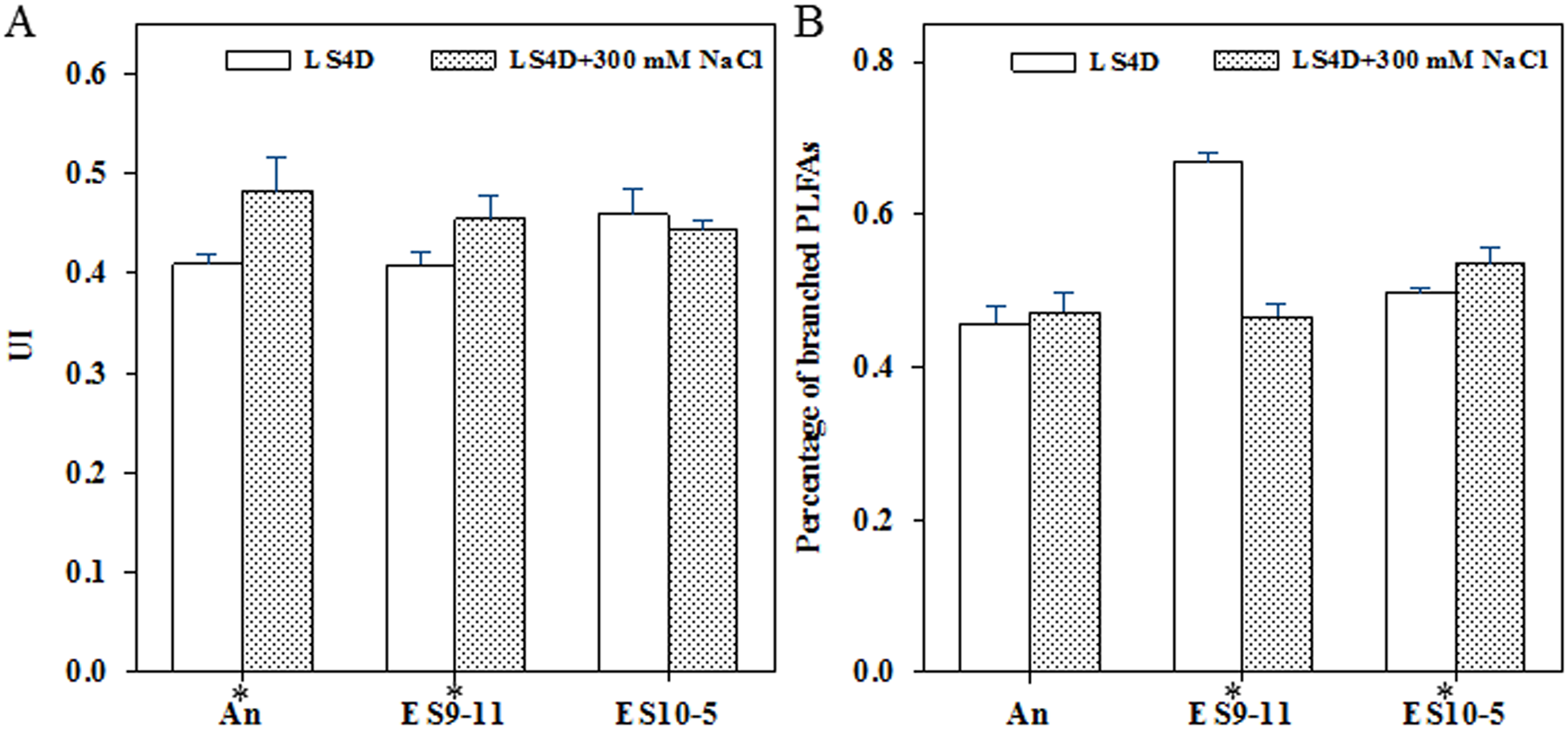

Supplement: FIG S2 [file mbo006173579sf2.tif]

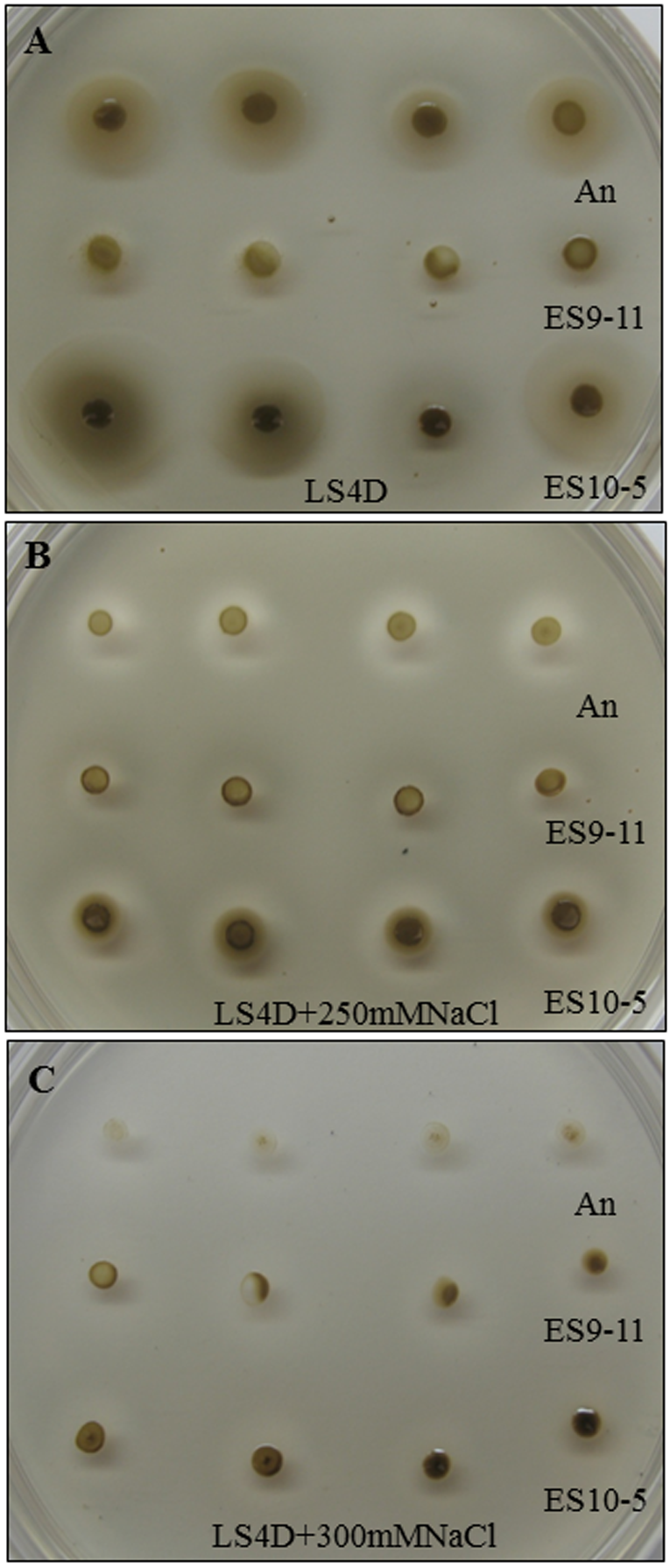

Supplement: FIG S3 [file mbo006173579sf3.tif]

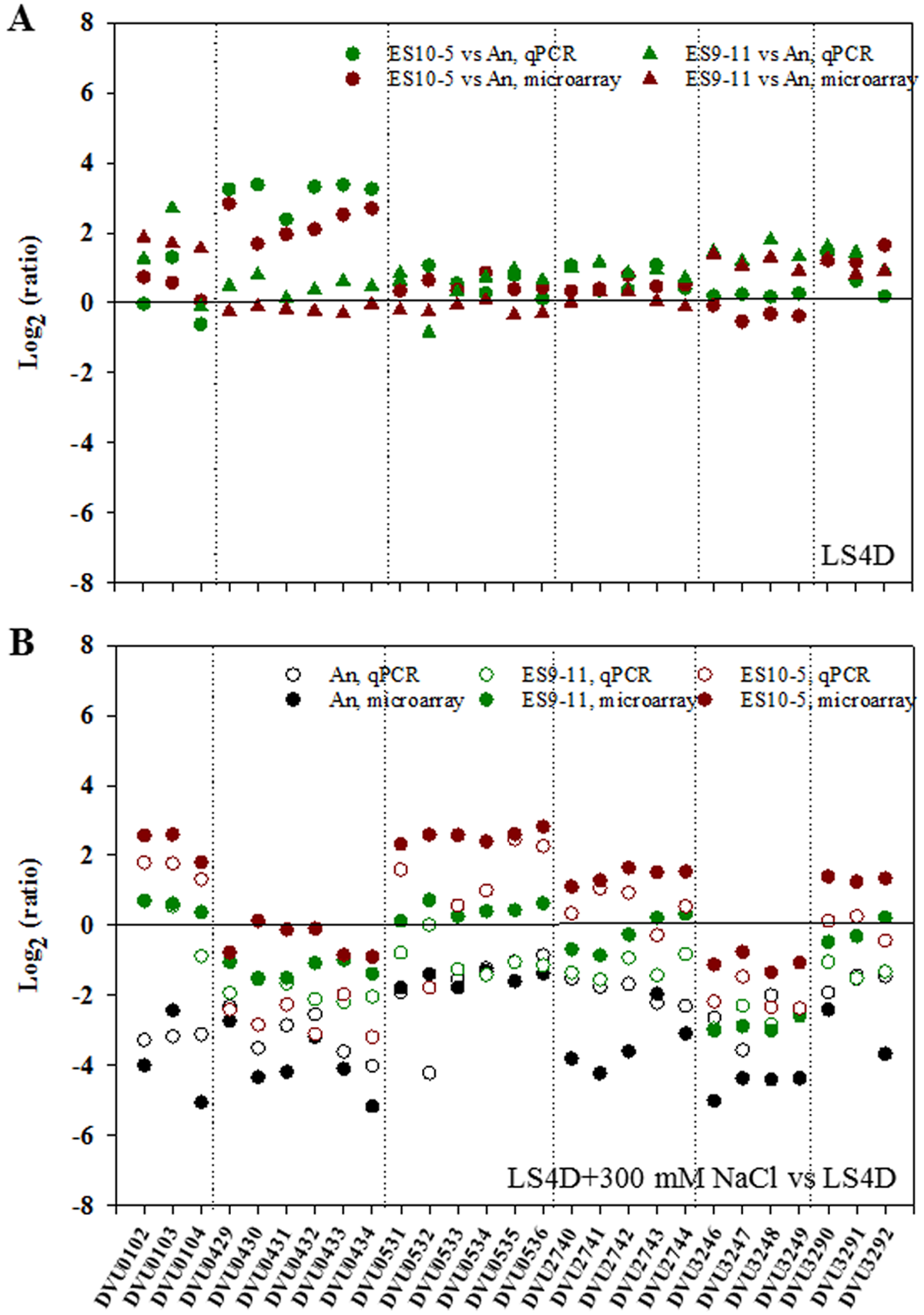

Supplement: FIG S4 [file mbo006173579sf4.tif]
