# Supplementary material for: Key Metabolites and Mechanistic Changes for Salt Tolerance in an Experimentally Evolved Sulfate-Reducing Bacterium, Desulfovibrio vulgaris
Source: mBio. 2017 Nov 14;8(6):e01780-17. doi: 10.1128/mBio.01780-17 (PMC5686539; doi:10.1128/mBio.01780-17)
Supplement: Supplementary file 2 [file mbo006173579st1.docx]

Table S1. Genes affected by the big deletion of 37390 bp from 233143 to 270532				
Gene name	Annotation	Location	COG(s)	COG function category
DVU_tRNA-Pseudo-1	pseudogene tRNA with anticodon TTT	233143-233215 (-)	-	-
DVU0189	P4 family phage/plasmid primase	233752-235437 (+)	COG3378	R
DVU0190	hypothetical protein	235434-235646 (-)	-	S
DVU0191	hypothetical protein	235704-237263 (+)	COG0358	L
DVU0192	adenine specific DNA methyltransferase, putative	237390-238748 (+)	COG0863	LK
DVU0193	hypothetical protein	238741-239472 (+)	COG1428	F
DVU0194	terminase, large subunit, putative	239489-241447 (+)	COG5525	R
DVU0195	hypothetical protein	241444-241662 (+)	-	S
DVU0196	hypothetical protein	242446-242586 (+)	-	S
DVU0197	lambda family phage portal protein	242590-244290 (+)	COG5511	R
DVU0198	minor capsid protein C, degenerate	244274-245593 (+)	COG0616	OU
DVU0199	hypothetical protein	245603-245986 (+)	-	S
DVU0200	major head protein	245998-247002 (+)	-	S
DVU0201	hypothetical protein	247011-247331 (+)	-	S
DVU0202	holin	247334-247822 (+)	COG4824	R
DVU0203	hypothetical protein	247843-248391 (+)	COG3926	R
DVU0204	putative lipoprotein	248409-248819 (+)	-	S
DVU0205	hypothetical protein	248812-248979 (+)	-	S
DVU0206	hypothetical protein	248982-249575 (+)	-	S
DVU0207	hypothetical protein	249572-249898 (+)	-	S
DVU0208	hypothetical protein	249895-250452 (+)	-	S
DVU0209	hypothetical protein	250436-250627 (+)	-	S
DVU0210	tail sheath protein, putative	250631-252094 (+)	COG4386	R
DVU0211	tail tube protein, putative	252144-252509 (+)	-	S
DVU0212	hypothetical protein	252509-252769 (+)	-	S
DVU0213	hypothetical protein	252969-254459 (+)	COG3941	R
DVU0214	tail/DNA circulation protein, putative	254468-255667 (+)	COG4228	R
DVU0215	tail protein, putative	255790-256923 (+)	COG4379	R
DVU0216	phage baseplate assembly protein V, putative	256925-257563 (+)	COG4384	S
DVU0217	tail protein, putative	257565-258041 (+)	COG4381	S
DVU0218	tail protein, putative	258050-259111 (+)	COG3299	S
DVU0219	tail protein, putative	259120-259746 (+)	COG3778	S
DVU0220	tail fiber protein, putative	259739-260755 (+)	-	S
DVU0221	tail fiber assembly protein, putative	260765-261208 (+)	-	S
DVU0222	hypothetical protein	261220-261549 (+)	-	S
DVU0223	hypothetical protein	261649-261831 (+)	COG1724	N
DVU0224	hypothetical protein	261875-262270 (+)	COG1598	S
DVU0225	hypothetical protein	262334-262510 (+)	-	S
DVU0226	hypothetical protein	262657-263796 (-)	-	S
DVU0227	hypothetical protein	263799-264329 (-)	COG3013	S
DVU0228	hypothetical protein	264667-264765 (+)	-	S
DVU0230	transcriptional regulator cII, putative	265802-266266 (+)	-	S
DVU0231	hypothetical protein	266347-266571 (+)	-	S
DVU0232	hypothetical protein	266564-266812 (+)	COG0376	P
DVU0233	hypothetical protein	266937-267239 (-)	-	S
DVU0234	hypothetical protein	267982-268305 (+)	-	S
DVU0235	hypothetical protein	268308-268871 (+)	-	S
DVU0236	phage integrase family site specific recombinase	269283-270425 (-)	COG0582	L

R: general function prediction only; S: function unknown; N: cell motility; P: inorganic ion transport and metabolism; L: replication, recombination and repair; K: transcription; O: posttranslational modification, protein turnover, chaperones; U: intercellular tracking, secretion, and vesicular transport.

(+): positive strand; (-): negative strand
