# Supplementary material for: Key Metabolites and Mechanistic Changes for Salt Tolerance in an Experimentally Evolved Sulfate-Reducing Bacterium, Desulfovibrio vulgaris
Source: mBio. 2017 Nov 14;8(6):e01780-17. doi: 10.1128/mBio.01780-17 (PMC5686539; doi:10.1128/mBio.01780-17)
Supplement: Supplementary file 3 [file mbo006173579st2.docx]

Table S2. Details of the genes deleted in plasmid due to the deletion from 51979b to 157737				
Gene name	Annotation	Location	COG(s)	COG function category
DVUA0043	polysaccharide deacetylase family protein	51965-52816(+)		G
DVUA0044	hypothetical protein	52878-53855(+)		S
DVUA0045	aminotransferase	53852-55468(+)		M
DVUA0046	glycosyl transferase, group 2 family protein	55465-56913(+)		M
DVUA0047	hypothetical protein	56989-58305(+)		S
DVUA0048	exopolysaccharide production protein	58360-59757(+)	COG3307	M
DVUA0049	polysaccharide biosynthesis family protein	59761-61230(+)		M
DVUA0050	hypothetical protein	61246-62502(+)		S
DVUA0051	glycosyl transferase, group 1 family protein	62499-63887(+)		M
DVUA0052	hypothetical protein	64069-64737(+)		S
DVUA0053	hypothetical protein	64988-65575(+)		S
DVUA0054	glycosyl transferase, group 1 family protein	65572-66972(+)		M
DVUA0055	hypothetical protein	66969-67922(+)		S
DVUA0056	hypothetical protein	67912-68532(+)		S
DVUA0057	sigma-54 dependent transcriptional regulator/response regulator	68687-70117(+)		T
DVUA0058	BNR/Asp-box repeat-containing protein	70158-72893(+)		S
DVUA0059	hypothetical protein	72938-74314(+)		S
DVUA0060	hypothetical protein	74453-75838(+)		S
DVUA0061	hypothetical protein	75835-77187(+)		S
DVUA0062	hypothetical protein	77184-78479(+)		S
DVUA0063	Orn/DAP/Arg family decarboxylase	78536-80092(+)		E
DVUA0064	hypothetical protein	80843-82885(+)		S
DVUA0065	sensor histidine kinase	82882-84918(+)	COG642	T
DVUA0066	patatin family phospholipase	85372-86784(+)	COG1752	S
DVUA0067	hypothetical protein	87157-88281(-)		S
DVUA0068	hypothetical protein	88333-90315(-)		S
DVUA0069	GtrA family protein, selenocysteine-containing	90871-92028(-)		S
DVUA0070	hypothetical protein	92091-94022(-)		S
DVUA0071	glycosyl transferase, group 1/2 family protein	94115-96271(-)		M
DVUA0072	glycosyl transferase, group 1 family protein	96400-97737(-)	COG438	M
DVUA0073	asparagine synthase (glutamine-hydrolyzing)	97846-100515(-)	COG0367	E
DVUA0074	sulfotransferase family protein	100606-101295(-)		EFHI
DVUA0075	radical SAM domain-containing protein	101383-102789(-)		EFHI
DVUA0076	ABC transporter ATP-binding protein	102804-104198(-)	COG1134	GM
DVUA0077	ABC transporter permease	104230-105114(-)		GM
DVUA0078	hypothetical protein	105230-105388(-)		S
DVUA0079(*cysC*)	adenylyl-sulfate kinase	105582-106268(+)	COG529	P
DVUA0080	glycosyl transferase, group 2 family protein	106366-107247(+)		M
DVUA0081	glycosyl transferase, group 1/2 family protein	107247-109361(+)	COG438	M
DVUA0082	phage integrase family site specific recombinase	109666-110697(-)	COG4973	L
DVUA0083	hypothetical protein	110806-110934(+)		S
DVUA0084	AbrB family transcriptional regulator	111110-111301(+)		T
DVUA0085	hypothetical protein	111356-114886(+)		S
DVUA0086	response regulator	114998-116089(-)	COG3437	T
DVUA0087	sensory box histidine kinase	116475-119588(-)		T
DVUA0088	hypothetical protein	119777-120796(-)		S
DVUA0089	hypothetical protein	121289-121906(-)		S
DVUA0090	hypothetical protein	122486-123358(+)		S
DVUA0091(*katA*)	catalase	123495-124952(+)	COG0753	P
DVUA0092	hypothetical protein	125171-125650(-)		S
DVUA0093	chromate transport family protein	125839-127176(-)	COG2059	P
DVUA0094(*chrB*)	chrB protein (chromate resistance exported protein)	127186-128190(-)		P
DVUA0095	hypothetical protein	128594-129079(+)		S
DVUA0096	major facilitator superfamily protein	129163-130374(+)	COG2814	G
DVUA0097	radical SAM domain-containing protein	130707-132206(+)		EFHI
DVUA0098	dehydrogenase	132351-133400(-)	COG0371	C
DVUA0099	HAMP domain-containing protein	133570-135753(-)	COG2208	T
DVUA0100(*flrC*)	sigma-54 dependent transcriptional regulator	136112-137839(-)	COG2204	T
DVUA0101(*flhB*)	hrpY/hrcU family type III secretion protein	137887-138927(-)	COG1377	N
DVUA0102(*escT*)	type III secretion inner membrane protein	138924-139739(-)		U
DVUA0103(*invX*)	HrpO family type III secretion protein	139746-140006(-)	COG4794	U
DVUA0104 (*sctV*)	type III secretion inner membrane protein HrcV family	140328-142421(-)		U
DVUA0105	hypothetical protein	143145-143519(-)		S
DVUA0106	YopN family type III secretion target protein	143522-144646(-)		U
DVUA0107	hypothetical protein	144885-144989(+)		S
DVUA203	hypothetical protein	145353-145655(+)		S
DVUA0108	hypothetical protein	145580-146200(+)		S
DVUA0109	LcrH/SycD family type III secretion system chaperone	146209-146709(+)		U
DVUA0110	type III secretion system target, YopB family	146706-147713(+)		U
DVUA0111	type III secretion system protein IpaC family	147662-148558(+)		U
DVUA201	hypothetical protein	148611-149066(+)		S
DVUA0112	type III secretion system protein YscC family	149369-151243(+)	COG1450	U
DVUA0113	YscD family type III secretion protein	151240-153048(+)		U
DVUA0114	hypothetical protein	153041-153250(+)		S
DVUA0115	type III secretion system protein YscF family	153276-153527(+)		U
DVUA0116	hypothetical protein	153722-154087(+)		S
DVUA0117(*escJ*)	type III secretion lipoprotein	154970-155785(+)	COG4669	U
DVUA200	hypothetical protein	155792-156715(+)		S
DVUA0118	type III secretion system protein	156694-157311(+)		U
DVUA0119(*sctN*)	type III secretion system ATPase	157639-158955(+)	COG1157	NU

S: function unknown; U: intracellular trafficking, secretion, and vesicular transport; P: inorganic ion transport and metabolism; N: cell motility;
M: cell wall/membrane/envelope biogenesis; T: signal transduction mechanisms; C: energy production and conversion; transport and metabolism of carbohydrate (G), amino acid (E), nucleotide (F), coenzyme (H), or lipid (I); (+): positive strand; (-): negative strand
