# Supplementary material for: Key Metabolites and Mechanistic Changes for Salt Tolerance in an Experimentally Evolved Sulfate-Reducing Bacterium, Desulfovibrio vulgaris
Source: mBio. 2017 Nov 14;8(6):e01780-17. doi: 10.1128/mBio.01780-17 (PMC5686539; doi:10.1128/mBio.01780-17)
Supplement: Supplementary file 4 [file mbo006173579st3.docx]

Table S3. PCR primers used to confirm the mutations with Sanger sequencing			
**Targeted gene**	**Targeted mutation position**	**Forward primer**	**Reverse primer**
DVU0071	88598	CGTGCCAGCTTCTGGTAGAC	GTTCACATCGGAGGCAATCT
DVU_tRNA-Pseudo-1	233143~270532	CGCAGAGTTGGATGAATGGT	CGGTCTCGACGTTGTGTGT
DVU0201	233143~270533	AGGCCGAGTTCTTCTCCAAG	ACAAAGCCCAGCACGTAGTC
DVU0236	233143~270534	GCTCTTCTCGGCCCTTGTA	ATTGGCTCACAAACGCCTAC
DVU0829	919173	CACCAACGACCTCATCCAGT	GACATAGCTGTCGCGGAAGT
DVU1204	1296277	CAGGCGAAGTGAAGGATTTC	GACCGAAGACCTGCTTGATG
DVU1260	1347079	CAGCTTGAGGGTACCCATGT	AGCAGGGACATTGCTGAAGT
DVU1530	1599083	TTGTAGATTCGGGCGAAGAC	AACAGTTCGCCGTTGATCTC
DVU1663	1743449	TGTCATCCATCTGGAAGTGG	GGGGACTCTCACCGTAATGA
DVU1838	1909403	GTGCTCATGACCTCCGAAAT	AGACCATCGAGTTCGAGGAA
DVU1862	1930032	GTTGCCTGTGGAATTGTCG	GTTCCCGGTGGACGTAGAT
DVU2001*	2083308	AAACATCAGGCTTGGACGAC	GTTCCCGAGGTGAAGTTGAG
DVU2298	2392375	CACCATGAGCACCATAGCC	GCTACCTGTCGACGGAACTG
DVU2349	2444619	GCATGAGCATGGTCTTTCTG	CAGGAGTATCTCCGCTTCCA
DVU2394	2497747	TTTTTGCGCAATGAGAACAG	AACACGTTGATGCGATGGTA
DVU2396	2499697	GCTGGAGAGACTCAGCATCG	AGATGATGCGCAAATCCTTC
DVU2571	2685414	GAAGCTTGCAACACTGCTCA	AGGAGAACGAACACCAGCAG
DVU2950*	3056212	CTCTTCATCGAACCGGAGTC	ATGCGGTACTCTGCCTCGTA
DVU3022*	3140596	ATCACCAAACGCAAGGAGAC	ATGAGGTCGGTGAAGGTCAG
DVU3151	3304807	TGCGTCGGTAAAATCCCTAC	AGAGTCTGCCGGGAGTGAC
*: pre-existing mutations in the DvH ancestor			
