# Supplementary material for: Key Metabolites and Mechanistic Changes for Salt Tolerance in an Experimentally Evolved Sulfate-Reducing Bacterium, Desulfovibrio vulgaris
Source: mBio. 2017 Nov 14;8(6):e01780-17. doi: 10.1128/mBio.01780-17 (PMC5686539; doi:10.1128/mBio.01780-17)
Supplement: Supplementary file 5 [file mbo006173579st4.docx]

Table S4. Metabolites detected by Q-TOF

RT	ID	m/z	Ion	Ion Formula	Peak area											
					An-C		An-T		ES9-11-C		ES9-11-T		ES10-5-C		ES10-5-T	
					Average	Stdev	Average	Stdev	Average	Stdev	Average	Stdev	Average	Stdev	Average	Stdev
28.335		154.0963	(M+H)+	C7 H12 N3 O	24,725	9,109	6,064	1,967	56,112	18,366	7,859	2,031	39,006	6,862	15,798	3,386
26.606		554.2148	(M+2H)+2	> limit	128,424	66,544	2,909	713	169,186	113,704	17,224	8,701	166,396	102,716	4,983	1,671
25.563		575.2245	(M+2H)+2	> limit	2,187,575	234,645	95,295	25,447	4,051,729	736,089	637,121	53,595	2,974,300	331,931	343,564	60,408
24.849		184.1054	(M+H)+	C8 H14 N3 O2	1,063	800	12,718	5,128	1,571	1,178	84,034	47,535	4,518	5,628	178,852	46,783
24.684		276.1540	(M+H)+	C7 H18 N9 O3	290	255	0	0	434	391	1,754	125	352	279	8,310	2,688
24.272		539.7077	(M+2H)+2	> limit	12,115	2,426	71	98	101,043	39,554	4,961	1,291	50,729	7,900	2,153	1,106
24.245		304.1612	(M+H)+	C11 H22 N5 O5	518	352	545	443	404	298	7,082	1,096	782	534	23,148	7,433
22.433	Piperazineethane sulfonic acid	195.0812	(M+H)+	C6 H15 N2 O3 S	1,322,347	418,597	1,995,537	695,786	1,374,564	564,310	1,651,593	316,699	1,430,536	452,362	1,698,778	202,925
21.911		134.0811	(M+H)+	C5 H12 N O3	1,808	714	974	915	1,913	911	13,756	6,135	245	549	60,292	15,673
22.241		539.7061	(M+2H)+2	> limit	13,280	6,621	73	100	38,486	22,053	737	338	16,943	13,028	126	89
21.472	citrulline	176.1026	(M+H)+	C6 H14 N3 O3	160,516	25,240	9,970	7,275	516,958	76,468	442,094	121,226	409,182	67,024	404,934	33,529
21.362		560.7057	(M+2H)+2	> limit	161,312	30,673	5,659	2,183	497,126	106,115	34,961	9,264	241,241	32,802	8,618	2,427
20.896	glutamine	147.0772	(M+H)+	C5 H11 N2 O3	533,403	59,486	69,854	32,166	1,289,909	185,159	2,108,450	708,664	800,708	69,699	2,511,635	334,293
20.703	C7 H13 N O5	192.0874	(M+H)+	C7 H14 N O5	1,952	285	3,651	1,441	1,796	1,445	71,316	10,919	3,539	1,923	55,734	10,058
20.456	alanine	90.0548	(M+H)+	C3 H8 N O2	88,800	6,225	102,428	20,421	1,031,693	89,253	1,052,130	247,669	98,242	13,330	124,471	30,430
20.346	glutamate	148.0599	(M+H)+	C5 H10 N O4	2,470,277	205,878	1,379,306	215,502	4,310,259	339,633	11,925,348	1,924,531	5,723,211	606,441	18,106,314	1,939,014
20.209	threonine	120.0638	(M+H)+	<none>	27,794	8,201	2,580	312	121,352	25,231	85,573	31,938	81,065	15,311	17,104	4,176
20.072		173.0249	(M+H)+	C4 H11 Cl2 N2 O	66,234	37,914	7,938	5,966	60,011	39,328	1,941	1,355	76,535	34,494	5,011	2,975
20.044	methenamine	141.1132	(M+H)+	C6 H13 N4	63,106	124,229	3,334,028	1,167,795	4,219	6,422	173,723	116,245	175,645	239,842	133,916	84,249
19.962		320.0738	(M+H)+	C10 H14 N3 O9	28,047	13,695	64	144	157,054	47,606	5,069	3,350	226,311	31,187	12,105	7,816
19.77	AMP	348.0713	(M+H)+	C12 H10 N7 O6	181,151	213,867	7,530	4,142	240,459	310,188	152,843	121,728	483,507	567,868	620,829	209,251
19.44		263.1002	(M+H)+	C9 H11 N8 O2	85	77	2,615	2,379	26	57	14,496	6,965	26	58	9,968	5,832
18.287		258.1724	(M+H)+	C14 H20 N5	10,891	5,506	20,172	14,204	12,225	4,732	17,550	6,666	17,518	7,295	12,034	6,810
18.205		476.1849	(M+H)+	C19 H26 N9 O4 S	35,243	3,937	26,917	34,857	24,278	8,120	12,776	5,770	22,282	2,434	8,375	5,046
18.205		286.2883	(M+H)+	C16 H36 N3 O	21,633	14,639	254,343	201,374	17,536	8,314	42,472	25,344	30,682	9,876	27,093	31,380
17.985	valine	118.0855	(M+H)+	C5 H12 N O2	40,720	7,389	10,490	6,475	338,597	110,694	236,033	69,825	99,504	26,440	169,280	107,327
17.601		713.0721	(M+H)+	C21 H29 O25 S	66,418	19,563	576,471	124,298	64,063	51,484	190,587	59,155	68,046	23,444	129,606	16,096
16.723		895.4329	(M+2H)+2	> limit	89	122	0	0	1,296	859	0	0	79	122	0	0
16.064		102.1277	(M+H)+	C6 H16 N	593,765	56,853	1,061,987	82,886	545,078	49,074	618,410	31,092	577,230	47,351	547,740	42,809
16.5	isoleucine	132.1018	(M+H)+	C6 H14 N O2	16,885	4,203	9,731	6,429	170,251	56,915	69,601	22,480	65,276	10,713	41,665	23,591
16.064	leucine	132.1017	(M+H)+	C6 H14 N O2	61,143	15,844	10,810	6,679	362,972	111,871	179,812	50,507	230,073	73,345	280,908	158,885
15.268		326.3204	(M+H)+	<none>	9,459	4,118	10,953	5,558	109,249	21,777	82,844	29,133	11,649	5,824	453	342
15.185		328.2980	(M+H)+	C23 H38 N	17,007	2,852	17,797	2,535	82,445	15,424	66,141	8,833	13,555	1,887	189	164
15.158		215.2119	(M+H)+	C12 H27 N2 O	16,966	2,972	9,304	2,513	55,329	12,309	54,545	10,452	16,629	3,378	135	131
15.15		342.3092	(M+H)+	C15 H36 N9	5,073	1,291	8,355	1,372	50,524	9,426	30,752	2,689	4,311	615	43	59
15.075		606.2772	(M+H)+	C30 H48 N5 S4	2,561	754	9,987	2,669	473	277	16,910	5,165	479	236	4,117	2,389
14.636		130.1575	(M+H)+	C8 H20 N	398,578	70,720	815,375	302,708	352,040	37,651	327,327	106,690	362,695	141,481	309,119	73,870
14.114		496.3019	(M+H)+	C21 H38 N9 O5	80,451	22,606	2,473	880	27,434	5,912	13,877	1,615	13,961	8,351	5,612	3,301
13.977		440.2775	(M+H)+	C13 H30 N17 O	1,035,993	275,735	122,168	75,687	614,612	137,715	311,675	64,744	985,294	350,582	285,959	209,163
13.95		498.3193	(M+H)+	C18 H44 N9 O5 S	147,010	41,874	7,115	1,184	14,876	1,713	30,804	6,600	10,639	5,230	8,625	4,848
13.84		454.2930	(M+H)+	C14 H32 N17 O	1,118,593	227,506	279,236	163,327	809,904	210,845	499,789	115,256	884,080	263,572	376,936	244,716
13.813		466.2945	(M+H)+	C22 H48 N3 O S3	1,346,454	255,354	227,715	121,799	746,809	191,377	533,137	129,208	1,345,458	436,784	388,102	268,792
13.62		482.3270	(M+H)+	C19 H44 N7 O7	917,747	126,125	151,586	78,326	152,958	36,749	244,155	60,179	193,736	67,149	100,135	63,989
13.593		480.3098	(M+H)+	C16 H34 N17 O	568,286	75,531	107,087	44,622	227,631	70,140	236,305	60,342	304,896	106,490	120,836	76,212
13.209		818.4938	(M+H)+	> limit	9,240	3,537	6,453	2,529	1,766	765	61,722	6,554	3,209	1,057	62,394	35,243
13.181		256.3004	(M+H)+	C17 H38 N	130,223	32,412	212,164	19,415	137,108	20,560	133,152	16,449	170,134	34,115	117,787	20,012
13.154		844.5136	(M+H)+	> limit	22,728	8,894	24,710	4,566	3,079	1,584	99,594	10,036	6,444	3,087	94,234	51,297
12.687		512.3697	(M+2H)+2	> limit	573	293	55,901	24,035	266	199	4,134	1,369	492	412	17,317	1,781
11.644	hypoxanthine	137.0458	(M+H)+	C5 H5 N4 O	5,538,051	694,949	26,061	26,071	4,430,858	986,810	552,670	199,059	4,061,203	1,878,927	2,766,710	1,304,597
10.985		330.3355	(M+H)+	C16 H40 N7	5,554,016	701,858	6,227,853	2,695,412	7,187,906	775,783	6,695,310	1,138,815	7,546,239	2,501,693	4,337,232	650,551
10.875		164.9350	(M+H)+	C3 H2 Cl N2 S2	337	685	385,481	293,736	46	103	10,714	23,957	1,946	4,350	22,965	30,792
10.793		358.3688	(M+H)+	C22 H48 N O2	9,830,026	1,147,336	12,574,662	5,335,974	11,893,371	1,485,476	11,543,308	1,696,027	13,852,739	4,048,381	8,380,317	583,432
9.228	adenine	136.0616	(M+H)+	C5 H6 N5	691,519	190,248	185,767	120,883	284,773	78,706	893,784	402,941	1,146,862	437,925	813,601	430,403
9.173		678.5058	(M+H)+	C33 H68 N5 O9	1,147,322	171,736	845,190	182,298	1,389,594	318,612	1,059,217	265,006	1,991,854	318,233	1,524,591	304,036
8.596		690.5014	(M+H)+	C32 H68 N9 O5 S	2,010,420	240,744	1,755,935	255,025	3,744,518	246,894	2,301,790	335,114	2,975,132	497,794	2,338,841	396,846
8.075		702.5063	(M+H)+	C26 H60 N19 O4	875,408	128,816	654,859	79,460	1,684,960	182,594	1,316,127	131,752	1,577,533	240,792	971,723	153,741
7.91		688.4909	(M+H)+	C26 H54 N23	343,197	53,561	280,487	28,333	1,004,087	92,972	644,424	69,978	675,663	114,430	379,636	52,907
7.416		738.5323	(M+H)+	C36 H80 N7 S4	46,185	5,671	37,130	6,502	109,561	8,487	81,431	21,207	84,087	18,553	70,039	27,401
7.388		195.1241	(M+H)+	C9 H15 N4 O	292,208	105,318	342,011	298,258	267,163	98,653	292,022	127,126	280,838	152,073	144,792	64,561
7.388		239.1503	(M+H)+	C11 H19 N4 O2	117,862	27,523	164,133	131,053	110,430	39,507	112,424	38,755	108,378	51,763	69,238	29,779
7.306		156.1367	(M+H)+	C9 H18 N O	226,843	120,650	430,833	164,082	322,886	193,819	208,959	44,799	301,632	132,482	136,523	61,927
4.863		718.5453	(M+H)+	C35 H72 N7 O8	547,976	44,931	664,441	106,030	249,211	45,600	489,271	74,187	279,315	50,313	333,183	88,362
4.725		690.5126	(M+H)+	C26 H56 N23	816,994	128,705	673,595	131,002	1,370,292	192,819	868,224	118,104	1,072,414	281,746	867,194	259,395
4.698		358.3687	(M+H)+	C22 H48 N O2	178,198	258,271	1,754,633	439,150	127,894	135,332	585,771	332,029	593,112	326,185	295,928	264,136
4.67		466.2931	(M+H)+	C25 H36 N7 O2	62,275	24,960	8,608	4,008	21,805	4,985	17,236	6,451	47,414	27,907	12,650	5,484
4.643		702.5021	(M+H)+	C36 H64 N9 O5	380,745	66,330	250,804	53,354	674,413	81,864	481,577	104,828	559,960	134,411	356,941	105,399
4.616		356.3507	(M+H)+	C22 H46 N O2	53,348	9,844	270,988	67,741	145,517	125,841	120,491	46,003	112,555	102,102	66,457	27,097
4.588		514.2856	(M+H)+	C22 H44 N O12	221,747	147,064	126,461	98,740	157,405	79,033	146,273	53,146	123,060	64,791	37,850	20,598
4.588		540.4279	(M+H)+	C31 H58 N O6	173,834	88,777	274,076	58,810	182,488	35,461	198,522	50,725	220,492	79,899	189,499	44,909
4.506		658.5109	(M+H)+	C34 H68 N5 O7	135,714	62,577	84,786	38,015	113,455	56,897	246,552	118,098	210,818	165,410	58,176	35,712
4.286		738.5319	(M+H)+	C36 H80 N7 S4	313,659	49,548	189,359	55,593	620,781	191,748	378,422	138,872	443,786	149,239	338,227	151,612
4.176		394.3558	(M+H)+	C22 H44 N5 O	1,431,781	447,671	919,531	344,838	1,172,435	511,913	969,519	563,870	1,091,704	390,570	304,925	83,280
3.984		682.1880	(M+3H)+3	> limit	39,150	13,812	115,836	61,440	40,508	23,107	51,322	26,319	30,583	7,651	100,722	51,806
3.957		739.8968	(M+3H)+3	> limit	32,549	12,525	144,143	85,910	38,122	18,820	69,974	33,208	25,216	6,030	141,236	79,040
3.929		778.5776	(M+3H)+3	> limit	61,664	26,964	169,925	93,652	47,254	27,285	89,461	54,175	39,345	17,997	165,225	100,561
3.902		836.6226	(M+3H)+3	> limit	6,270	5,350	79,312	50,414	7,917	7,367	34,153	24,876	3,163	2,906	73,484	34,240
3.874		391.2817	(M+H)+	C20 H35 N6 O2	5,119,470	1,568,852	9,245,079	2,451,561	5,230,835	838,876	6,330,189	1,182,823	5,642,328	1,904,748	5,086,659	1,415,043
3.874		654.3316	(M+H)+	C27 H40 N15 O5	1,204,248	509,497	1,911,240	542,385	1,159,054	288,153	1,251,271	381,036	1,330,635	485,127	1,181,630	297,093

Metabolites detected by positive mode are listed. C: LS4D; T: LS4D+300 mM NaCl
