# Supplementary material for: Key Metabolites and Mechanistic Changes for Salt Tolerance in an Experimentally Evolved Sulfate-Reducing Bacterium, Desulfovibrio vulgaris
Source: mBio. 2017 Nov 14;8(6):e01780-17. doi: 10.1128/mBio.01780-17 (PMC5686539; doi:10.1128/mBio.01780-17)
Supplement: Supplementary file 6 [file mbo006173579st5.docx]

Table S5 Selected genes and gene specific primers for reverse transcription real time PCR

Target gene	Forward primer sequence	Reverse primer sequence	Product size (bp)
DVU3246	GACGCATTGTCAGCAACGTA	AACCTGTCTATGGCGTCCTC	115
DVU3247	TCGAATCCGTGGACATCAC	ATAGGGTTCCGGCTCGAT	116
DVU3248	GTGAAACGACAGGGCGTAAC	AGTGATGCGAAGTTGCTCAG	116
DVU3249	CGGCTTTCTCCGTGACTATC	GACCCTGTCGATGACGATG	118
DVU3290	GTTCTTCATCGAAGGCGTGT	TCGGACTGGTAGCCCATGTA	114
DVU3291	GTGCGGCCTACAACATCTC	CCATGATGGGAATCTCGTTC	114
DVU3292	ATCCTCATCCACGGCAAC	CGGGTTCTGCTTGGTCAT	114
DVU1068	GTGTTGCGTCTGCGTTTC	GAAGCTTGTCGGGTCCTTC	117
DVU1069	CCCATCCTGTATGCCACTCT	AGGTGCAGGGCGAGAAAC	116
DVU1070	CTGTCTCTCGACCTTGTGGA	TGTATTCGGCGAGAATGTCA	118
DVU2740	TCAAGTCCATCATGCGTCTG	CAGCTTGCGTACCACATCAT	116
DVU2741	GGCCTCAATTCCGTAGATGT	AGCGACATCACCACCTTC	113
DVU2742	GGGAACGCTGTTCTCACTGT	GTGTCGACGCTCATGATTTC	114
DVU2743	GGCAGATAGGGGGATTCTTC	GTCTGTAGGCCACCCGTTC	117
DVU2744	AACCGCTACGGCTACATCC	CATCTTGCGGACATCCTTCT	114
DVU0102	GCTGGTCATCCTGATGCTCT	ACGTCCTTGACCACGTCCT	114
DVU0103	CTCGAACGCACGGGTATC	AGCAGGATGTCGGGTTCC	116
DVU0104	CTGACAGGGGTGAGATTTCG	GTTCAGGGCCTCGGTCTT	116
DVU0429	TCTGCAAGAGCTGCCAGATA	CATCGACGCATACAGAGCAG	114
DVU0430	TGGTCATCTCCACCAACATC	GTCATCAGGGCACGGATTT	115
DVU0431	CTGACCTTTGACGGTCTGGT	ATGCCTTCTTCTGGATGGTG	115
DVU0432	TCAACCATCGCAACAAGAAG	TGTAGCACTCGCGGAACA	117
DVU0433	TCGAAGTGCTCATCGACAAC	TGACGTACAGCCAAAGGATG	114
DVU0434	CCCTGTCTGCTCTCGGAAT	GTAGAAAAGCAGCGCGAAAT	116
DVU2378	CCACCATGCGTGAATACCT	AGGGCAGACTCTCGTAGCC	118
DVU2086	GAGTGGTTCTGGCAGAGGAC	CAACATCCCCGTGTCCAT	116
DVU0525	CAGATGAAGAACGGCACCTA	CCATATTCCCCCACCAGACT	115
DVU0531	GCAGGACTCGTGCAACTACA	TAGTTGTGCTCGCGGTTG	114
DVU0532	CCTGCATGTGGGCGACTA	CGACATGAAGAACAGCACGA	115
DVU0533	CACGAGTTCATGCTCCACAC	ATTTGCGGATCTTCTCGTCA	115
DVU0534	CTGGTGCTGACCATCTTCG	CAGGAACGACGAGTACCACA	114
DVU0535	CTGCAACGAAGTGAACAAGC	GCGTTGTACCTGTTGACCAC	117
DVU0536	ATGACATCCCCGAAAAGGTC	CCAGCTTGTCTTCACCGATA	117
DVU0094	CCGTGTTGCTGTGGTTCAC	CATGAAGCGGAGACAGCAT	117
DVU0862	TCATCAACGAACTCGACAGC	AGCTTCAGGTTGGCACTGAG	115
DVU2444	ACACCATCTCCACCCAGTCT	TGTTCTCAAGGCGGTTCTG	115
