# Supplementary material for: Key Metabolites and Mechanistic Changes for Salt Tolerance in an Experimentally Evolved Sulfate-Reducing Bacterium, Desulfovibrio vulgaris
Source: mBio. 2017 Nov 14;8(6):e01780-17. doi: 10.1128/mBio.01780-17 (PMC5686539; doi:10.1128/mBio.01780-17)
Supplement: Supplementary file 7 [file mbo006173579st6.docx]

Table S6. Expression of genes affected by the big deletions in chromosome or plasmid DNA in LS4D

		ES9-11		ES10-5		
Gene name	Annotation	Log_2R	Z-score	Log_2R	Z-score	location
DVU0190	hypothetical protein	-0.18	-0.30	**-2.53**	**-4.26**	deletion in the chromosome of ES10-5
DVU0193	hypothetical protein	-0.68	-0.84	**-6.17**	**-7.77**	(233143 to 270532)
DVU0194	terminase, large subunit, putative	-0.53	-0.77	**-2.66**	**-3.95**	
DVU0195	hypothetical protein	-0.48	-0.79	**-2.16**	**-3.52**	
DVU0213	conserved domain protein	-0.41	-0.47	**-6.35**	**-7.60**	
DVU0220	tail fiber protein, putative	-0.40	-0.46	**-5.37**	**-6.25**	
DVU0221	tail fiber assembly protein, putative	-0.34	-0.34	**-5.09**	**-5.22**	
DVU0223	conserved hypothetical protein	0.06	0.04	**-6.15**	**-4.85**	
DVU0224	conserved hypothetical protein	-0.11	-0.11	**-5.32**	**-5.51**	
DVU0226	hypothetical protein	-0.44	-0.39	**-6.26**	**-5.88**	
DVU0227	conserved hypothetical protein	-0.57	-0.42	**-4.24**	**-3.17**	
VIMSS11399146	hypothetical protein	-0.01	-0.01	**-7.01**	**-6.51**	
DVU0230	transcriptional regulator cII, putative	-0.21	-0.33	**-1.65**	**-2.55**	
DVU0231	hypothetical protein	-0.83	-1.11	**-6.55**	**-8.97**	
DVU0232	hypothetical protein	-0.86	-1.07	**-3.90**	**-4.93**	
VIMSS11399147	hypothetical protein	-0.77	-1.07	**-4.07**	**-4.56**	
DVU0234	hypothetical protein	-0.84	-0.87	**-4.44**	**-4.66**	
DVU0235	hypothetical protein	-0.78	-0.83	**-7.01**	**-7.61**	
DVU0236	site-specific recombinase, phage integrase family	-0.30	-0.34	**-4.07**	**-4.57**	
DVUA0043	polysaccharide deacetylase family protein	0.42	0.79	**-1.63**	**-3.06**	deletion in the plasmid of ES10-5
DVUA0044	conserved hypothetical protein	0.16	0.29	**-2.18**	**-4.03**	(51979 to 157737)
DVUA0047	hypothetical protein	0.04	0.08	**-1.57**	**-2.78**	
DVUA0048	exopolysaccharide production protein, putative	0.26	0.50	**-2.55**	**-4.79**	
DVUA0049	polysaccharide biosynthesis family protein	0.18	0.33	**-2.13**	**-3.89**	
DVUA0051	glycosyl transferase, group 1 family protein	0.18	0.34	**-3.76**	**-7.15**	
DVUA0052	conserved hypothetical protein	0.04	0.07	**-4.52**	**-7.71**	
DVUA0053	conserved hypothetical protein	0.32	0.54	**-2.38**	**-4.15**	
DVUA0054	glycosyl transferase, group 1 family protein	0.02	0.04	**-1.07**	**-1.73**	
DVUA0055	membrane protein, putative	0.14	0.27	**-1.84**	**-3.48**	
DVUA0057	sigma-54 dependent transcriptional regulator/response regulator	0.20	0.36	**-1.82**	**-3.19**	
DVUA0058	BNR/Asp-box repeat protein	0.37	0.59	**-3.79**	**-5.99**	
DVUA0060	membrane protein, putative	0.05	0.09	**-1.19**	**-2.16**	
DVUA0061	membrane protein, putative	0.16	0.29	**-1.71**	**-3.05**	
DVUA0062	conserved domain protein	0.25	0.44	**-2.14**	**-3.81**	
DVUA0063	Orn/DAP/Arg family decarboxylase	0.30	0.55	**-1.86**	**-3.38**	
DVUA0063.1	hypothetical protein	0.33	0.59	**-4.07**	**-7.39**	
DVUA0064	gene conserved domain protein	0.66	0.84	**-3.24**	**-4.39**	
DVUA0065	sensor histidine kinase	0.34	0.57	**-2.27**	**-3.80**	
DVUA0066	phospholipase, patatin family	0.07	0.11	**-4.37**	**-7.08**	
DVUA0067	membrane protein, putative	-0.64	-0.60	**-6.64**	**-6.32**	
DVUA0068	membrane protein, putative	-0.20	-0.16	**-4.94**	**-3.96**	
VIMSS11399254	hypothetical protein	-0.19	-0.19	**-5.04**	**-5.10**	
DVUA0069	GtrA family protein, selenocysteine-containing	-0.29	-0.27	**-6.80**	**-6.52**	
DVUA0070	conserved domain protein	-0.22	-0.21	**-5.86**	**-5.60**	
DVUA0071	glycosyl transferase, group 1/2 family protein	-0.53	-0.46	**-5.37**	**-4.77**	
DVUA0072	glycosyl transferase, group 1 family protein	-0.48	-0.35	**-7.33**	**-5.51**	
DVUA0073	asparagine synthase (glutamine-hydrolyzing), putative	-0.52	-0.52	**-5.92**	**-5.83**	
DVUA0074	sulfotransferase family protein	-0.53	-0.38	**-7.00**	**-5.07**	
DVUA0075	radical SAM domain protein	-0.35	-0.28	**-7.66**	**-6.29**	
DVUA0076	ABC transporter, ATP-binding protein	-0.41	-0.36	**-8.82**	**-7.86**	
DVUA0077	ABC transporter, permease protein	-0.55	-0.59	**-6.30**	**-6.72**	
DVUA0079(*cysC*)	adenylylsulfate kinase	-0.17	-0.14	**-5.01**	**-4.23**	
DVUA0080	glycosyl transferase, group 2 family protein	-0.22	-0.19	**-6.57**	**-5.88**	
DVUA0081	glycosyl transferase, group 1/2 family protein	-0.04	-0.05	**-3.73**	**-4.77**	
DVUA0082	site-specific recombinase, phage integrase family	-0.28	-0.37	**-2.87**	**-4.03**	
DVUA0084	transcriptional regulator, AbrB family	-0.52	-0.56	**-5.31**	**-5.98**	
DVUA0085	conserved hypothetical protein	-0.11	-0.14	**-7.36**	**-9.18**	
DVUA0086	response regulator	-0.15	-0.15	**-6.44**	**-6.94**	
VIMSS11399255	hypothetical protein	-0.51	-0.44	**-6.45**	**-5.55**	
DVUA0087	sensory box histidine kinase	-0.02	-0.02	**-3.49**	**-4.51**	
DVUA0088	conserved hypothetical protein	-0.05	-0.07	**-5.64**	**-7.53**	
DVUA0089	hypothetical protein	-0.22	-0.40	**-1.90**	**-3.45**	
DVUA0090	membrane protein, putative	0.00	0.00	**-2.94**	**-4.74**	
DVUA0091	catalase	-0.18	-0.16	**-4.87**	**-4.29**	
DVUA0092	hypothetical protein	**-2.22**	**-2.51**	**-6.76**	**-7.75**	
DVUA0093	chromate transport family protein	-0.07	-0.10	**-1.20**	**-1.88**	
DVUA0094(*chrB*)	chrB protein	0.16	0.25	**-2.19**	**-3.46**	
VIMSS11399257	hypothetical protein	-0.25	-0.42	**-3.37**	**-5.68**	
DVUA0096	major facilitator superfamily protein	0.43	0.79	**-1.76**	**-3.35**	
DVUA0097	radical SAM domain protein	-0.35	-0.40	**-3.67**	**-4.21**	
DVUA0098	dehydrogenase, putative	0.27	0.30	**-5.97**	**-7.28**	
DVUA0099	HAMP domain protein	-0.39	-0.39	**-6.45**	**-6.52**	
DVUA0100(*flrC*)	sigma-54 dependent transcriptional regulator	-0.20	-0.21	**-5.14**	**-5.42**	
DVUA0101(*flhB*)	type III secretion protein, hrpY/hrcU family	-0.35	-0.43	**-4.91**	**-6.09**	
DVUA0102(*escT*)	type III secretion inner membrane protein	0.09	0.13	**-2.07**	**-3.08**	
DVUA0103(*invX*)	type III secretion protein, HrpO family	-0.01	-0.01	**-4.90**	**-7.15**	
DVUA0104(*sctV*)	type III secretion inner membrane protein, HrcV family	-0.26	-0.34	**-3.97**	**-5.18**	
VIMSS11399261	hypothetical protein	-0.30	-0.39	**-4.14**	**-5.57**	
DVUA0105	conserved hypothetical protein	-0.28	-0.38	**-4.45**	**-6.26**	
DVUA0106	type III secretion target, YopN family	-0.29	-0.33	**-5.03**	**-5.90**	
DVUA203	hypothetical protein	-0.39	-0.37	**-4.72**	**-4.46**	
DVUA0108	hypothetical protein	-0.66	-0.61	**-5.65**	**-5.41**	
DVUA0109	type III secretion system chaperone, LcrH/SycD family	-0.34	-0.30	**-6.65**	**-6.00**	
DVUA0110	type III secretion system target, YopB family	-0.31	-0.32	**-5.97**	**-6.40**	
DVUA0111	type III secretion system protein, IpaC family, putative	-0.32	-0.33	**-4.22**	**-4.47**	
DVUA201	hypothetical protein	-0.29	-0.26	**-3.96**	**-3.62**	
DVUA0112	type III secretion system protein, YscC family	-0.24	-0.26	**-2.90**	**-3.07**	
DVUA0113	type III secretion protein, YscD family	-0.52	-0.48	**-5.03**	**-4.82**	
DVUA0115	type III secretion system protein, YscF family	-0.44	-0.42	**-6.75**	**-6.71**	
DVUA0116	conserved hypothetical protein	-0.30	-0.28	**-4.55**	**-4.34**	
VIMSS11399264	hypothetical protein	-0.28	-0.31	**-6.79**	**-7.93**	
DVUA0117(*escJ*)	type III secretion lipoprotein	-0.47	-0.60	**-4.88**	**-6.44**	
DVUA200	hypothetical protein	-0.24	-0.24	**-5.21**	**-5.39**	
DVUA0118	type III secretion protein, YopL family	-0.21	-0.21	**-4.60**	**-4.82**	
DVUA0119(*sctN*)	type III secretion system ATPase	-0.19	-0.22	**-3.60**	**-4.37**	
DVUA0119.1	hypothetical protein	-0.23	-0.24	**-2.75**	**-3.03**	immediate downstream of the deletion
DVUA0120	type III secretion protein, putative	-0.53	-0.45	**-3.18**	**-2.71**	in plasmid of ES10-5, to the last
DVUA0121	type III secretion system protein, YopQ family	-0.38	-0.48	**-1.96**	**-2.56**	gene of the affected operon
DVUA0122(*yscR*)	type III secretion system protein, YscR family	-0.36	-0.45	**-1.84**	**-2.34**	
DVUA0123	anti-anti-sigma factor	-0.14	-0.14	**-2.43**	**-2.67**	
DVUA0124	sigma factor serine-protein kinase	-0.24	-0.28	**-1.97**	**-2.36**	
DVUA0125(*slt*)	transglycosylase, SLT family	-0.22	-0.30	**-1.12**	**-1.58**	
DVU1779	hypothetical protein	**-6.58**	**-9.06**	0.06	0.06	deletion in the chromosome of ES9-11
DVU1780	conserved hypothetical protein	**-7.78**	**-8.39**	0.06	0.04	(1842155 to 1849967)
DVU1781	conserved hypothetical protein	**-4.94**	**-3.91**	0.66	0.82	
DVU1782	iron-sulfur cluster-binding protein	**-6.69**	**-6.09**	-0.05	-0.04	
DVU1783	cysteine-rich domain protein	**-7.25**	**-5.69**	-0.21	-0.17	
DVU1784	oxidoreductase, short-chain dehydrogenase/reductase family	**-7.16**	**-6.16**	0.14	0.11	
DVU1785	membrane protein, MarC family	**-5.13**	**-5.74**	0.03	0.02	
DVU1786	GGDEF domain protein	**-7.05**	**-6.44**	-0.22	-0.04	
