# Supplementary material for: Key Metabolites and Mechanistic Changes for Salt Tolerance in an Experimentally Evolved Sulfate-Reducing Bacterium, Desulfovibrio vulgaris
Source: mBio. 2017 Nov 14;8(6):e01780-17. doi: 10.1128/mBio.01780-17 (PMC5686539; doi:10.1128/mBio.01780-17)
Supplement: Supplementary file 8 [file mbo006173579st7.docx]

Table S7. High salinity induced gene expression changes					
		ES9-11		ES10-5	
Locus Tag	Annotation	Log_2R	Z-score	Log_2R	Z-score
**General stress response**					
DVU2986	phage shock protein C	**1.69**	**2.13**	0.98	1.00
DVU2987	hypothetical protein	-0.82	-0.28	0.18	0.17
DVU2988	phage shock protein A	**1.65**	**1.34**	0.79	0.53
DVU2441	HSP20 family protein	**2.18**	**1.68**	1.17	0.79
DVU2442	HSP20 family protein	**1.19**	**1.17**	0.61	0.46
**Replication, recombination and repair**					
DVUA0004	DNA-binding protein HU, putative	0.30	0.53	**1.65**	**1.63**
DVU3256(*mutM*)	formamidopyrimidine-DNA glycosylase	**-1.90**	**-3.47**	-0.65	-0.96
**General function or function unknown**					
DVU0308	hypothetical protein	**2.13**	**3.06**	**1.87**	**1.91**
DVU1971	hypothetical protein	**2.07**	**2.28**	**2.07**	**1.91**
DVU0065	hypothetical protein	**1.53**	**2.60**	**2.49**	**2.77**
DVU1092	sodium-dependent symporter family protein	**-1.92**	**-3.27**	**-1.90**	**-2.55**
DVU2345	hypothetical protein	**-2.38**	**-3.40**	**-2.97**	**-4.40**
DVU2429	hypothetical protein	**-1.63**	**-2.16**	**-1.45**	**-1.55**
DVU2431	hypothetical protein	**-2.29**	**-3.74**	**-2.03**	**-2.91**
DVU2676	hypothetical protein	**-1.49**	**-1.36**	**-2.21**	**-2.07**
DVU2626	hypothetical protein	**-1.84**	**-2.49**	**-1.57**	**-1.76**
DVU3298	hypothetical protein	**-1.11**	**-2.03**	**-2.65**	**-4.01**
DVU3299	hypothetical protein	-0.89	-1.72	**-3.56**	**-5.25**
DVU3300	hypothetical protein	**-2.25**	**-4.37**	**-4.06**	**-5.63**
DVU3301	hypothetical protein	**-1.64**	**-3.20**	**-4.35**	**-6.63**
DVU3302	hypothetical protein	**-1.77**	**-3.39**	**-4.44**	**-6.56**
DVU0616	hypothetical protein	**-1.29**	**-2.36**	**-1.18**	**-1.85**
DVU0617	hypothetical protein	**-1.68**	**-2.75**	**-2.13**	**-3.23**
DVU2789	Gpr1/Fun34/YaaH family protein	**-2.41**	**-4.27**	**-1.97**	**-2.60**
DVU0729	hypothetical protein DVU0729	**-1.00**	**-1.62**	**-1.92**	**-2.32**
DVU2631	hypothetical protein	-0.24	-0.47	**1.60**	**2.40**
DVU3130	hypothetical protein	-0.31	-0.49	**-2.03**	**-2.27**
DVU0475	membrane protein, putative, truncation	-0.65	-0.98	**-1.65**	**-1.55**
DVU3384(*zraP*)	zinc resistance-associated protein	**1.77**	**1.61**	0.09	0.08
DVU0383	hypothetical protein	**-1.50**	**-2.65**	-0.23	-0.35
DVU0614	hypothetical protein	**-1.62**	**-2.57**	-0.90	-0.96
DVU1991	hypothetical protein	**-1.72**	**-3.21**	-0.22	-0.37
DVU2187	hypothetical protein	**-1.70**	**-3.36**	-0.59	-1.14
DVU2625	hypothetical protein	**-1.63**	**-2.11**	-0.35	-0.42
DVU3166	alanyl-tRNA synthetase family protein,	**-1.60**	**-2.82**	-0.37	-0.45
DVU3255	CopG family transcriptional regulator	**-1.71**	**-2.82**	-0.84	-0.95
DVU0008	hypothetical protein DVU0008	**-1.75**	**-3.32**	-0.58	-1.02

Operons are separated by lines. Bold font indicates more than two folds of significant expression changes.
